# 9-Butyl-Harmol Exerts Antiviral Activity against Newcastle Disease Virus through Targeting GSK-3β and HSP90β

**DOI:** 10.1128/jvi.01984-22

**Published:** 2023-03-06

**Authors:** Chongyang Wang, Ting Wang, Ruochen Hu, Liuyuan Duan, Qili Hou, Yu Han, Jiangkun Dai, Wenbin Wang, Shanhui Ren, Haijin Liu, Xinglong Wang, Sa Xiao, Na Li, Junru Wang, Zengqi Yang

**Affiliations:** a College of Veterinary Medicine, Northwest A&F University, Yangling, China; b College of Chemistry and Pharmacy, Northwest A&F University, Yangling, China; c Poultry Institute, Shandong Academy of Agricultural Science, Jinan, China; d State Key Laboratory of Veterinary Etiological Biology, Lanzhou Veterinary Research Institute, Chinese Academy of Agricultural Sciences, Lanzhou, China; e Instrumental Analysis Center, Xi'an Jiaotong University, Xi’an, China; University of Kentucky College of Medicine

**Keywords:** Newcastle disease virus, β-carbolines, antiviral mechanism, GSK-3β, HSP90β

## Abstract

The paramyxoviruses represent a large family of human and animal pathogens that cause significant health and economic burdens worldwide. However, there are no available drugs against the virus. β-carboline alkaloids are a family of naturally occurring and synthetic products with outstanding antiviral activities. Here, we examined the antiviral effect of a series of β-carboline derivatives against several paramyxoviruses, including Newcastle disease virus (NDV), peste des petits ruminants virus (PPRV), and canine distemper virus (CDV). Among these derivatives, 9-butyl-harmol was identified as an effective antiviral agent against these paramyxoviruses. Further, a genome-wide transcriptome analysis in combination with target validation strategies reveals a unique antiviral mechanism of 9-butyl-harmol through the targeting of GSK-3β and HSP90β. On one hand, NDV infection blocks the Wnt/β-catenin pathway to suppress the host immune response. 9-butyl-harmol targeting GSK-3β dramatically activates the Wnt/β-catenin pathway, which results in the boosting of a robust immune response. On the other hand, NDV proliferation depends on the activity of HSP90. The L protein, but not the NP protein or the P protein, is proven to be a client protein of HSP90β, rather than HSP90α. 9-butyl-harmol targeting HSP90β decreases the stability of the NDV L protein. Our findings identify 9-butyl-harmol as a potential antiviral agent, provide mechanistic insights into the antiviral mechanism of 9-butyl-harmol, and illustrate the role of β-catenin and HSP90 during NDV infection.

**IMPORTANCE** Paramyxoviruses cause devastating impacts on health and the economy worldwide. However, there are no suitable drugs with which to counteract the viruses. We determined that 9-butyl-harmol could serve as a potential antiviral agent against paramyxoviruses. Until now, the antiviral mechanism of β-carboline derivatives against RNA viruses has rarely been studied. Here, we found that 9-butyl-harmol exerts dual mechanisms of antiviral action, with its antiviral activities being mediated by two targets: GSK-3β and HSP90β. Correspondingly, the interaction between NDV infection and the Wnt/β-catenin pathway or HSP90 is demonstrated in this study. Taken together, our findings shed light on the development of antiviral agents against paramyxoviruses, based on the β-carboline scaffold. These results present mechanistic insights into the polypharmacology of 9-butyl-harmol. Understanding this mechanism also deepens the host-virus interaction and reveals new drug targets for anti-paramyxoviruses.

## INTRODUCTION

Using natural products to develop drugs with potential therapeutic activity is a well-established and efficient strategy. β-carboline alkaloids, with a core indole structure and pyridine ring, are known to present a wide range of biological activities, including anticancer, anxiolytic, antiparasitic, antiviral, etc. (1). In past decades, β-carboline alkaloids have been found to exhibit excellent antiviral activity against both DNA and RNA viruses, including influenza A virus (IAV) (2), human papillomavirus (HPV) (3), human immunodeficiency virus (HIV) (4), herpes simplex virus (HSV) (5), dengue virus (DENV) (6), and enterovirus 71 (EV71) (7). However, the antiviral effect of β-carbolines against paramyxoviruses was rarely reported. Recently, we have found that several β-carboline derivatives have the potential to inhibit Newcastle disease virus (NDV), which belongs to the family Paramyxoviridae (8, 9). Therefore, it is of potential value to further screen antiviral agents against paramyxoviruses from β-carboline derivatives.

To date, only the target of β-carboline compounds against HSV has been reported. Bag et al. found that harmaline exhibits antiviral activity against both wild-type and clinical isolates of HSV-1. Harmaline directly binds to lysine-specific demethylase-1 (LSD1) and interferes with the recruitment of LSD1 by host cell factor-1 (HCF-1), which thereby leads to the suppression of viral immediate-early (IE)-transcriptional events (10). Nevertheless, it is a specific target protein that is only meaningful for HSV, which is a typical DNA virus. The antiviral mechanism and target of β-carboline compounds against paramyxoviruses are worth exploring.

As is known, virus proliferation is highly dependent upon host cells, presenting a double-edged sword. On one hand, some host factors can exhibit antiviral activity by which to combat virus infection, such as interferon (IFN) and interferon-stimulated genes (ISGs) (11). β-catenin, one of the key molecules in the Wnt/β-catenin pathway, could interact with IRF3 or TCF to promote IFN-β production, thereby leading to the upregulation of immune responses and regulation of viral proliferation (12, 13). On the other hand, some host factors can assist the synthesis of viral proteins and benefit virus proliferation, including molecular chaperones (14). HSP90, one of the most prominent chaperone members, could facilitate the synthesis and folding of viral proteins, thereby facilitating viral proliferation (15). Previous studies have shown that several β-carboline derivatives could interact with multiple host factors, including heat shock protein 90 (HSP90), dual-specificity tyrosine-regulated kinases 1A (DYRK1A), and glycogen synthase kinase-3β (GSK-3β), thereby regulating various signaling pathways in host cells (16, 17). Overall, the relationship between the host, virus, and β-carbolines is complicated and needs further research.

Here, 53 β-carboline alkaloid monomers and dimers were screened for antiviral activity against NDV. 9-butyl-harmol was identified as the most effective inhibitor of NDV proliferation, and it exhibited broad-spectrum antiviral activity against a range of RNA viruses. Further, we investigated the transcriptome data of host cells upon 9-butyl-harmol treatment. Based on these data, we attempted to dissect the antiviral mechanism of 9-butyl-harmol and identify the potential molecular target.

## RESULTS

### Screening of β-carboline alkaloid monomers and dimers with antiviral activity.

The structures of the 53 *β*-carboline derivatives that were used in this study are presented in Tables S1 and S2. In order to investigate the antiviral activities of β-carboline derivatives, the effects of compounds on cell viability were evaluated via CCK-8. β-carboline derivatives that decreased cell viability to less than 80% of that observed with DMSO-treated cells were considered to be cytotoxic and were eliminated from evaluation. Based on the cell viability, all β-carboline monomers were used at the concentration of 10 μM, except for 14. 39 β-carboline dimers and 14 were used at a concentration of 5 μM (Fig. S1A and S2A). The DF-1 cell line was used for the primary screening of the β-carboline derivatives. The standard of an active compound was one that exhibited a ≥50% reduction, compared to DMSO-treated cells. In the primary screening, 15 compounds were identified that met this standard via plaque assay and qRT-PCR (Fig. S1 and S2). Among them, 8 derivatives (6, 11–14, 18–20) exhibited superior activity, with their inhibitory rates being higher than 80%. 3 derivatives (6, 11, 14) showed excellent activity, with their inhibitory rates being higher than 99% (Fig. S1B). The IC_50_ values of these compounds were further examined via plaque assay, and their cytotoxicities were tested in parallel via CCK-8. As shown in Table 1, compared with the positive-control ribavirin, 15 derivatives exhibited superior activity, with their IC_50_ values being lower than 10 μM against F48E9. Two derivatives, namely, 11 and 14, displayed anti-NDV activity for F48E9, with their IC_50_ values being 0.49 μM and 0.69 μM, respectively. The selectivity index (SI) was calculated as the ratio CC_50_ / IC_50_. 3 derivatives (6, 11, 14) emerged with SI ≥ 10. Among them, 9-butyl-harmol (11) showed the strongest antiviral activity with the highest SI value (>102). In addition, 3 NDV strains were used to evaluate the antiviral activity of 15 derivatives. The results revealed that 15 derivatives displayed antiviral activity against various genotypes of NDV with IC_50_ values in the range of 0.58 to 8.87 μM (Table 2).

**TABLE 1:** Cytotoxicity and anti-NDV activity of *β*-carboline derivatives

No.	CC_50_^*a*^(μM)	IC_50_^*b*^(μM)	SI^*c*^
6	34.17 ± 2.85	1.27 ± 0.32	26.9
7	30.32 ± 4.34	6.57 ± 0.86	4.6
11	>50	0.49 ± 0.11	>102.0
12	>50	5.87 ± 1.92	>8.5
13	>50	8.02 ± 1.57	>6.2
14	8.52 ± 0.42	0.69 ± 0.13	12.3
30	8.72 ± 2.46	3.50 ± 0.84	2.5
31	8.62 ± 1.85	2.25 ± 1.55	3.8
32	9.49 ± 2.28	2.34 ± 0.13	4.1
33	9.66 ± 1.39	2.59 ± 0.68	3.7
34	32.45 ± 4.97	4.77 ± 0.72	6.8
35	28.24 ± 2.18	5.33 ± 1.29	5.3
37	30.66 ± 2.93	3.92 ± 0.87	7.8
48	31.35 ± 5.82	4.52 ± 1.45	6.9
49	30.82 ± 3.44	4.90 ± 0.73	6.3
Ribavirin		8.62 ± 1.44	

a DF-1 cells were treated with different concentrations of each compound at 37°C for 48 h. The cell viability was determined using a CCK-8 kit. CC_50_ values were calculated (mean ± SD).

b DF-1 cells that were infected with F48E9 (MOI = 0.01) were treated with different concentrations of each compound for 24 h. The supernatants were harvested to determine the virus production via plaque assay. IC_50_ values were calculated (mean ± SD).

c Selectivity index; SI = CC_50_ / IC_50_.

**TABLE 2:** IC_50_ values (μM) of 1-formyl-*β*-carboline derivatives against three genotypes of NDV^*a*^

No.	Blackbird/China/08(IX)	PPMV-1/SX-01/Ch/15(VI)	La sota (II)
6	0.82 ± 0.23	1.01 ± 0.39	1.45 ± 0.15
7	5.89 ± 0.85	6.21 ± 1.31	7.38 ± 0.81
11	0.63 ± 0.30	0.71 ± 0.26	0.58 ± 0.08
12	4.97 ± 0.67	6.25 ± 2.18	6.34 ± 0.59
13	7.22 ± 1.46	7.52 ± 1.74	8.87 ± 1.96
14	0.83 ± 0.21	0.64 ± 0.19	0.75 ± 0.12
30	2.81 ± 0.59	3.33 ± 1.28	2.51 ± 0.57
31	2.94 ± 0.83	2.09 ± 0.97	3.43 ± 1.15
32	2.75 ± 0.51	2.64 ± 0.46	2.85 ± 0.76
33	2.22 ± 0.22	2.91 ± 0.27	3.36 ± 1.53
34	5.12 ± 1.64	4.59 ± 1.98	6.32 ± 0.78
35	5.56 ± 0.48	6.07 ± 0.45	6.14 ± 1.21
37	4.34 ± 0.49	5.92 ± 1.95	4.81 ± 0.67
48	5.29 ± 0.90	4.84 ± 0.38	5.96 ± 0.70
49	5.47 ± 1.27	5.68 ± 0.74	6.63 ± 1.91
Ribavirin	10.41 ± 2.85	11.62 ± 2.13	7.97 ± 1.59

a DF-1 cells that were infected with different genotypes of NDV (MOI = 0.01) were treated with different concentrations of each compound for 24 h. The supernatants were harvested to determine the virus production via plaque assay. IC_50_ values were calculated (mean ± SD).

We attempted to determine whether these derivatives might exhibit broad antiviral activity beyond that against NDV infection. Vero cells that were infected with either peste des petits ruminants virus (PPRV) or canine distemper virus (CDV) were treated with 15 derivatives. As expected, the 15 derivatives also exerted antiviral activity against PPRV and CDV. All compounds caused a reduction of ≥50%. Inspiringly, 9-butyl-harmol (11) also possessed superior activity, with its inhibitory rate being higher than 99% (Fig. S3). Based on the above results, 9-butyl-harmol was chosen as the focus of the following studies.

### Antiviral effect and mode of action of 9-butyl-harmol against NDV.

**FIG 1:**
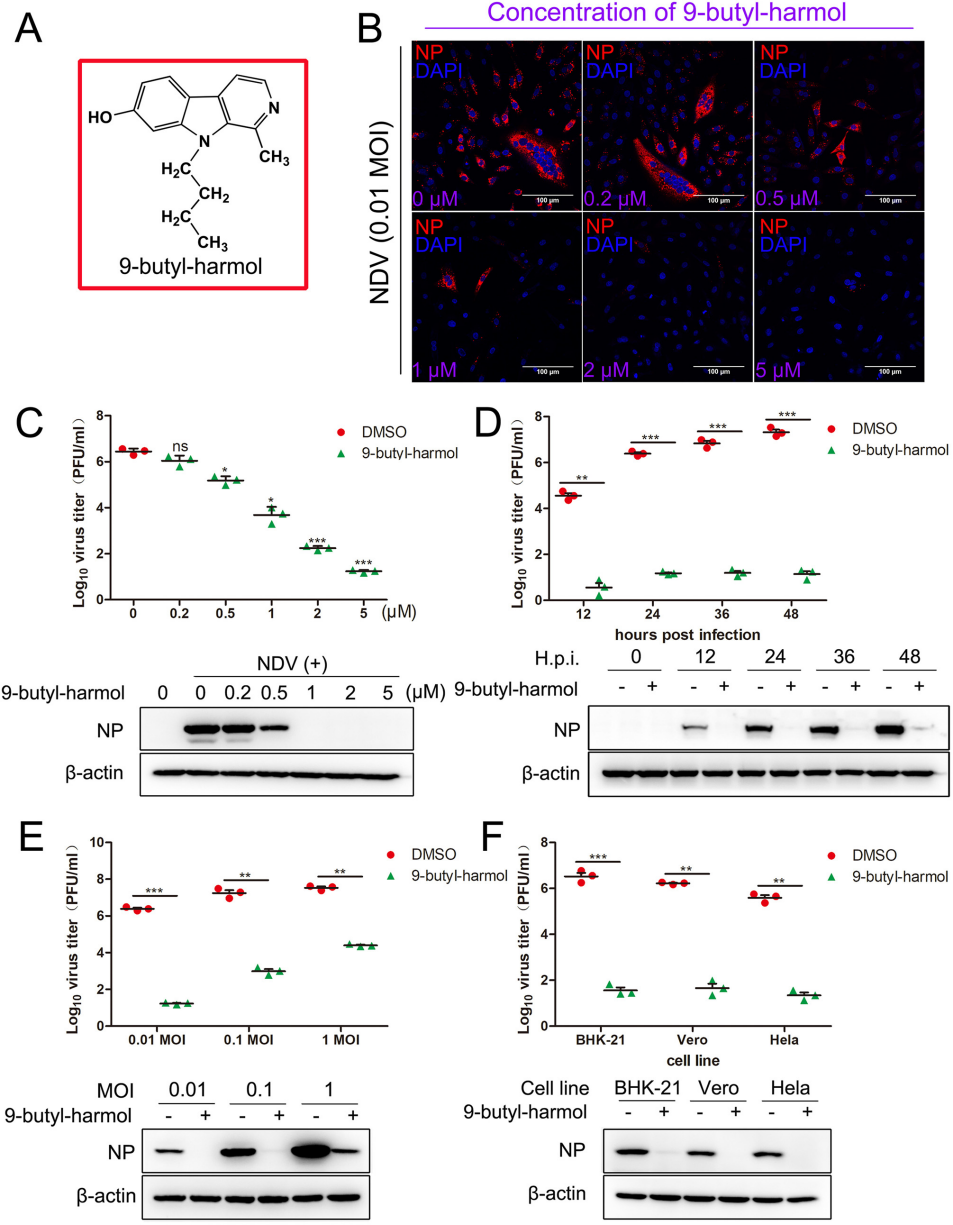
The antiviral effect of 9-butyl-harmol against NDV. (A) Structure of 9-butyl-harmol. (B and C) 9-butyl-harmol inhibits NDV proliferation in a dose-dependent manner. DF-1 cells were infected with F48E9 (0.01 MOI). The medium was changed to fresh medium containing 9-butyl-harmol at the indicated concentrations, and this was followed by 24 h of incubation. The viral protein was detected via immunofluorescence microscopy (B). The viral titer was assessed via plaque assay (C, upper panel). The viral protein was assessed via Western blotting (C, lower panel). The mean values ± SDs are shown (*n* = 3). Significance was assessed via two-tailed, unpaired Student’s *t* tests. ns, not significant; *, *P* < 0.05; ***, *P < *0.001. (D) The antiviral effect of 9-butyl-harmol against NDV at different time points. DF-1 cells were infected with F48E9 (0.01 MOI). The medium was changed to fresh medium containing 9-butyl-harmol (5 μM). The antiviral effect of 9-butyl-harmol at the indicated time points was assessed via plaque assay (upper panel) or Western blotting (lower panel). Mean values ± SDs are shown (*n* = 3). Significance was assessed via two-tailed, unpaired Student’s *t* tests. **, *P < *0.01; ***, *P < *0.001. (E) The antiviral effect of 9-butyl-harmol against NDV (various MOIs). DF-1 cells were infected with F48E9 (0.01, 0.1, or 1 MOI). The medium was changed to fresh medium containing 9-butyl-harmol (5 μM). The antiviral effect of 9-butyl-harmol against F48E9 was assessed via plaque assay (upper panel) or Western blotting (lower panel) at 24 h postinfection. Mean values ± SDs are shown (*n* = 3). Significance was assessed via two-tailed, unpaired Student’s *t* tests, **, *P < *0.01; ***, *P < *0.001. (F) The antiviral effect of 9-butyl-harmol in various cell lines. BHK-21, Vero, or HeLa cells were infected with F48E9 (0.01 MOI). The medium was changed to fresh medium containing 9-butyl-harmol (5 μM). The antiviral effect of 9-butyl-harmol in the indicated cell lines was assessed via plaque assay (upper panel) or Western blotting (lower panel) at 24 h postinfection. Mean values ± SDs are shown (*n* = 3). Significance was assessed via two-tailed, unpaired Student’s *t* tests. **, *P < *0.01; ***, *P < *0.001.

The structure of 9-butyl-harmol is presented in Fig. 1A. Several experiments were conducted to characterize the antiviral activity of 9-butyl-harmol against NDV (F48E9). From this point in the manuscript forward, all studies were conducted based on the F48E9 strain. As shown in Fig. 1B and C, treatment with 9-butyl-harmol significantly reduced the viral protein in a dose-dependent manner. The cytopathic effect that was induced by NDV infection was significantly alleviated upon the treatment of 9-butyl-harmol (Fig. 1B; Fig. S4A). In the presence of 5 μM 9-butyl-harmol, infectious virus particle production was reduced by 5.26 Log, compared to cells treated with equivalent volumes of DMSO (Fig. 1C). Additionally, the NDV mRNA markedly decreased along with the increasing concentration of 9-butyl-harmol (Fig. S4B). When the cells were treated with 9-butyl-harmol at 5 μM for 12 to 48 h, the infectious virus particle production and viral protein were both significantly inhibited (Fig. 1D). With high doses of NDV infection (1 MOI), 9-butyl-harmol also caused a significant reduction of 3.08 Log in infectious virus particle production (Fig. 1E). Furthermore, the inhibitory effect of 9-butyl-harmol was observed in three cell lines (Fig. 1F). Taken together, these results fully prove the antiviral effect of 9-butyl-harmol.

**FIG 2:**
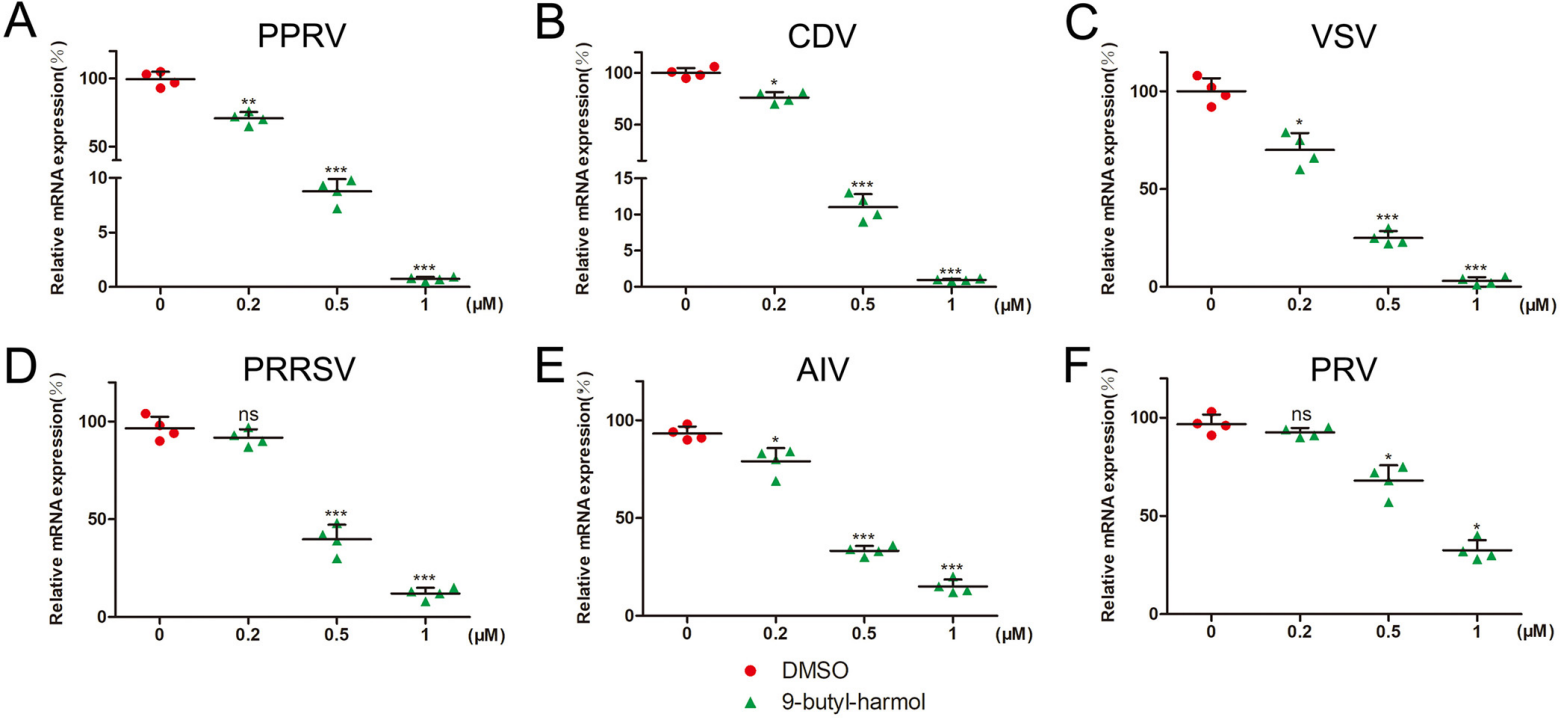
The 9-butyl-harmol exerts broad-spectrum antiviral activity. (A) Viral mRNA was assessed via qRT-PCR in Vero cells infected with PPRV and was treated with DMSO or 9-butyl-harmol for 24 h. Mean values ± SDs are shown (*n* = 4). Significance was assessed via two-tailed, unpaired Student’s *t* tests. **, *P < *0.01; ***, *P < *0.001. (B) Viral mRNA was assessed via qRT-PCR in Vero cells infected with CDV and was treated with DMSO or 9-butyl-harmol for 24 h. Mean values ± SDs are shown (*n* = 4). Significance was assessed via two-tailed, unpaired Student’s *t* tests. *, *P < *0.05; ***, *P < *0.001. (C) Viral mRNA was assessed via qRT-PCR in BHK-21 cells infected with VSV and was treated with DMSO or 9-butyl-harmol for 24 h. Mean values ± SDs are shown (*n* = 4). Significance was assessed with two-tailed, unpaired Student’s *t* tests. *, *P < *0.05: ***, *P < *0.001. (D) Viral mRNA was assessed via qRT-PCR in Marc-145 cells infected with PRRSV and was treated with DMSO or 9-butyl-harmol for 24 h. Mean values ± SDs are shown (*n* = 4). Significance was assessed via two-tailed, unpaired Student’s *t* tests. ns, not significant; ***, *P < *0.001. (E) Viral mRNA was assessed via qRT-PCR in A549 cells infected with AIV and was treated with DMSO or 9-butyl-harmol for 24 h. Mean values ± SDs are shown (*n* = 4). Significance was assessed via two-tailed, unpaired Student’s *t* tests. *, *P < *0.05; ***, *P < *0.001. (F) Viral mRNA was assessed via qRT-PCR in BHK-21 cells infected with PRV and was treated with DMSO or 9-butyl-harmol for 24 h. Mean values ± SDs are shown (*n* = 4). Significance was assessed with two-tailed, unpaired Student’s *t* tests. ns, not significant; *, *P < *0.05.

To examine the effect of 9-butyl-harmol on the proliferation of other viruses, cells were infected with PPRV, CDV, vesicular stomatitis virus (VSV), porcine reproductive and respiratory syndrome virus (PRRSV), avian influenza virus (AIV), and pseudorabies virus (PRV). As expected, with the increasing concentration of 9-butyl-harmol, all viral gene levels were decreased. In the presence of 1 μM 9-butyl-harmol, the mRNA of PPRV and CDV was reduced by 100-fold (Fig. 2A and B). Meanwhile, 9-butyl-harmol (1 μM) caused a >80% reduction in the mRNA expression levels of VSV, PRRSV, and AIV (Fig. 2C–E). However, under the same concentration, the mRNA of PRV (DNA virus) was only reduced by 66.83% (Fig. 2F). As shown in Table 3, 9-butyl-harmol displayed antiviral activities against these viruses with IC_50_ values in the range of 0.38 to 0.81 μM. These results suggest that 9-butyl-harmol exhibits broad antiviral activity.

**TABLE 3:** IC_50_ values (μM) of 9-butyl-harmol against different viruses^*a*^

Compound	PPRV	CDV	VSV	PRRSV	AIV	PRV
9-butyl-harmol	0.38 ± 0.06	0.40 ± 0.12	0.43 ± 0.16	0.54 ± 0.15	0.53 ± 0.18	0.81 ± 0.11

a Vero cells were infected with PPRV or CDV. BHK-21 cells were infected with VSV or PRV. Marc-145 cells were infected with PRRSV. A549 cells were infected with AIV. After that, the cells were treated with different concentrations of 9-butyl-harmol for 24 h. Viral mRNA was assessed via qRT-PCR. IC_50_ values were calculated.

**FIG 3:**
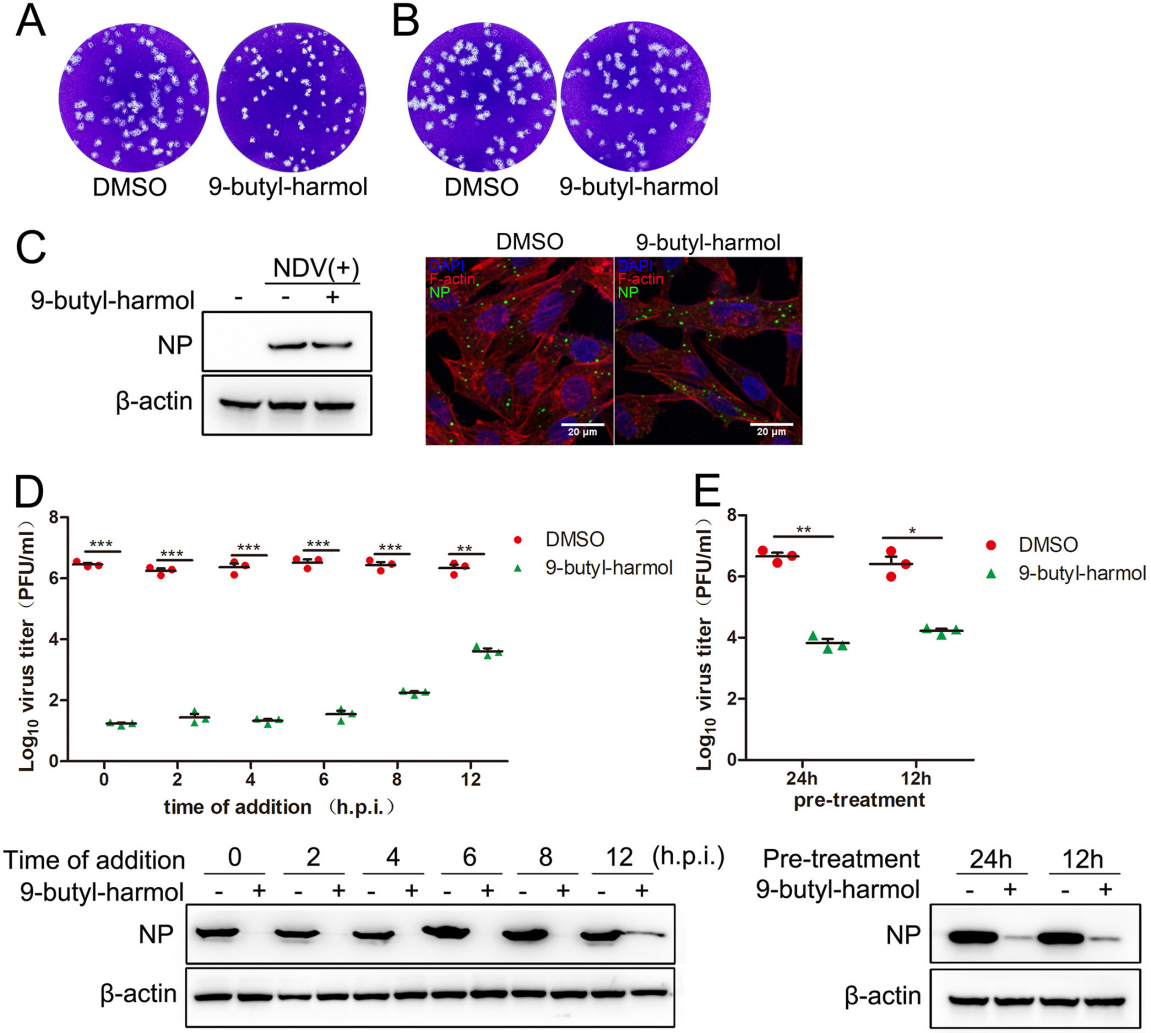
The mode of action of 9-butyl-harmol. (A) Infection of NDV in combination with 9-butyl-harmol has no effect on NDV adsorption. DF-1 cells were infected with F48E9 (100 PFU) and treated with 9-butyl-harmol (5 μM) at the same time. At 1 h postinfection, the cells were washed and covered with a medium containing methylcellulose. At 72 h postinfection, the virus yield was analyzed. (B) Pretreating cells with 9-butyl-harmol has no effect on NDV adsorption. DF-1 cells were treated with 9-butyl-harmol (5 μM) prior to F48E9 infection (100 PFU). At 1 h postinfection, the cells were washed and covered with a medium containing methylcellulose. At 72 h postinfection, the virus yield was analyzed. (C) 9-butyl-harmol has no effect on NDV penetration. DF-1 cells were infected with F48E9 (MOI = 5) at 4°C for 1 h. Unbound viruses were washed away by prechilled PBS. Next, the cells were supplemented with medium-containing 9-butyl-harmol (5 μM). At 4 h postinfection, the viral protein was measured by immunofluorescence (upper panel) or Western blotting (lower panel). (D) The antiviral effect of adding 9-butyl-harmol postinfection. 9-butyl-harmol (5 μM) was added at the indicated time points postinfection, and the antiviral effect was detected via plaque assay (upper panel) or Western blotting (lower panel) at 24 h postinfection. Mean values ± SDs are shown (*n* = 3). Significance was assessed via two-tailed, unpaired Student’s *t* tests. **, *P < *0.01; ***, *P < *0.001. (E) The antiviral effect of adding 9-butyl-harmol prior to infection. 9-butyl-harmol (5 μM) was added at the indicated time points prior to viral infection, and the antiviral effect was detected via plaque assay (upper panel) or Western blot (lower panel) at 24 h postinfection. Mean values ± SDs are shown (*n* = 3). Significance was assessed via two-tailed, unpaired Student’s *t* tests. *, *P* < 0.05; **, *P < *0.01.

To clarify which stage of the NDV (F48E9) life cycle is targeted by 9-butyl-harmol, we conducted a series of experiments. As shown in Fig. 3A and B, NDV adsorption was not inhibited by 9-butyl-harmol. Meanwhile, 9-butyl-harmol has no effect on the entry of NDV (Fig. 3C). A time-of-addition study showed that significant viral inhibition occurred when 5 μM 9-butyl-harmol was added to the cell culture prior to virus infection or within 12 h postinfection (Fig. 3D and E). These results suggest that the viral attachment and entry stages are not targeted by 9-butyl-harmol.

### Effect of 9-butyl-harmol on the transcriptome of DF-1 cells.

**FIG 4:**
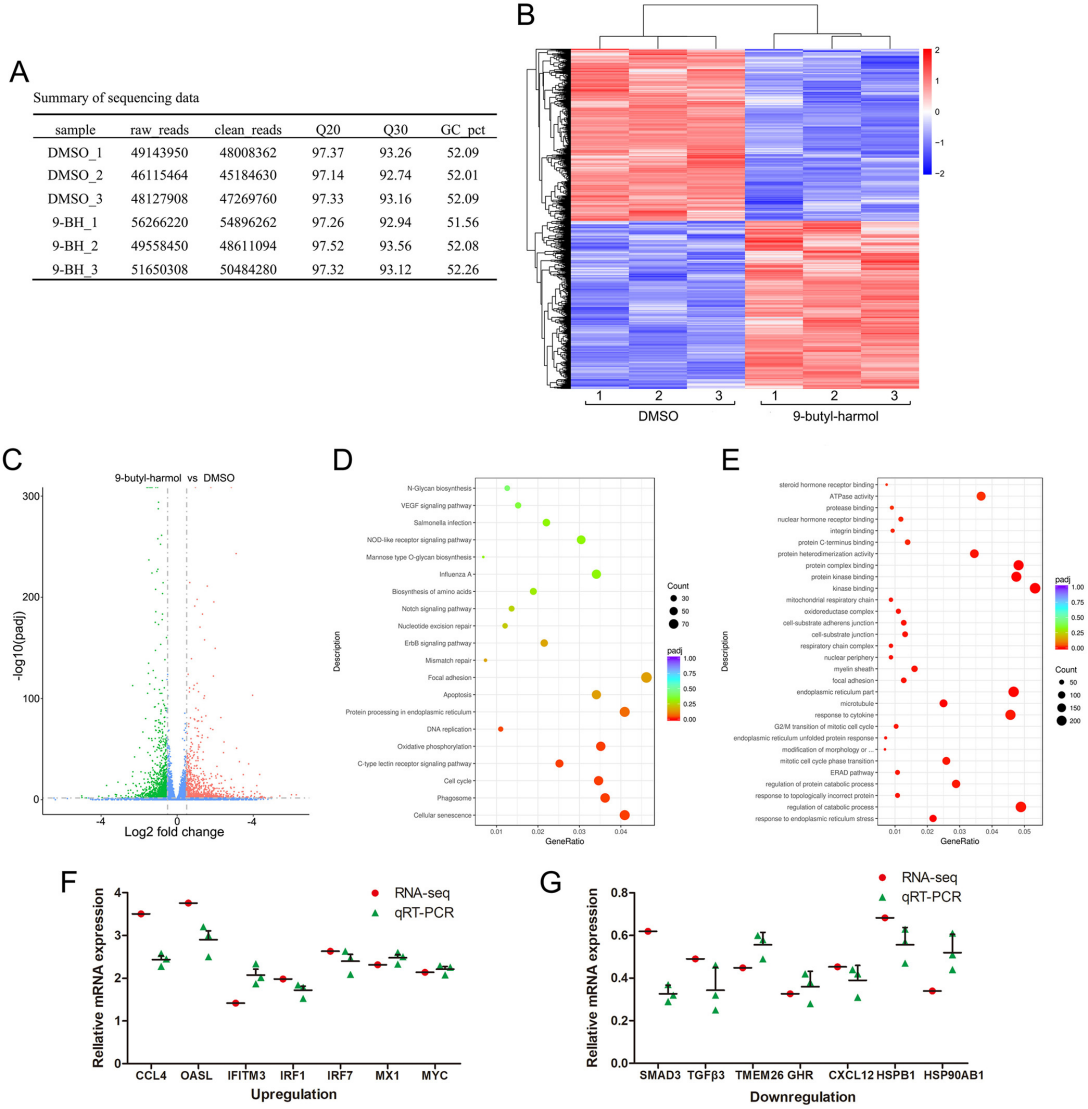
Analysis and validation of transcriptome data. (A) Summary of sequencing data. (B) The heat map of differentially expressed genes. (C) The volcano plots of differentially expressed genes. (D) Differentially expressed genes enriched in KEGG pathways. (E) GO analysis of differentially expressed genes. (F) Verification of upregulated genes in the RNA-seq via qRT-PCR. (G) Verification of downregulated genes in the RNA-seq via qRT-PCR.

To identify the target pathway or molecules of 9-butyl-harmol, two RNA libraries that were pooled from DMSO-treated to 9-butyl-harmol-treated DF-1 cells were constructed (three biological replicates per group). The library preparations were sequenced on an Illumina HiSeq 2500 platform, and 125 bp/150 bp paired-end reads were generated. Fig. 4A summarizes the sequencing data for each sample. Clean data (clean reads) were obtained by removing reads containing adapters and low-quality reads from the raw data. At the same time, the Q20, Q30, and GC content of the clean data were calculated. All of the downstream analyses were based on clean data that were of high quality. The transcripts were filtered by the thresholds of P-adj <0.02 and |log_2_ (fold change)| > 0.5. Under these criteria, 2,341 DEGs were identified, and these contained 1,145 upregulated genes and 1,196 downregulated genes (Fig. 4B and C).

The top 20 Kyoto Encyclopedia of Genes and Genomes (KEGG) pathways are shown in Fig. 4D. Many of the genes that are involved in the immune-related pathways, such as the influenza A signaling pathway (KEGG ID: gga05164), C-type lectin receptor signaling pathway (KEGG ID: gga04625), and NOD-like receptor signaling pathway (KEGG ID: gga04621), led us to question whether 9-butyl-harmol affects the immune response to modulate NDV proliferation. The top 30 Gene Ontology (GO) terms are shown in Fig. 4E, including the response to endoplasmic reticulum stress (GO ID: 0034976), response to topologically incorrect protein (GO ID: 0035966), and endoplasmic reticulum unfolded protein response (GO ID: 0030968), indicating that 9-butyl-harmol could affect protein folding. These data indicate that 9-butyl-harmol may affect protein folding to modulate virus infection. To verify the transcriptome data, qRT-PCR was performed to analyze the same RNA samples as were used in the transcriptome analysis for the 14 selected genes. As shown in Fig. 4F and G, all of the selected genes exhibited concordant trends, compared with the transcriptome data, supporting the claim that the DEGs that were identified by the transcriptome are reliable.

### 9-butyl-harmol activates the Wnt/β-catenin pathway by targeting GSK-3β to suppress NDV proliferation.

**FIG 5:**
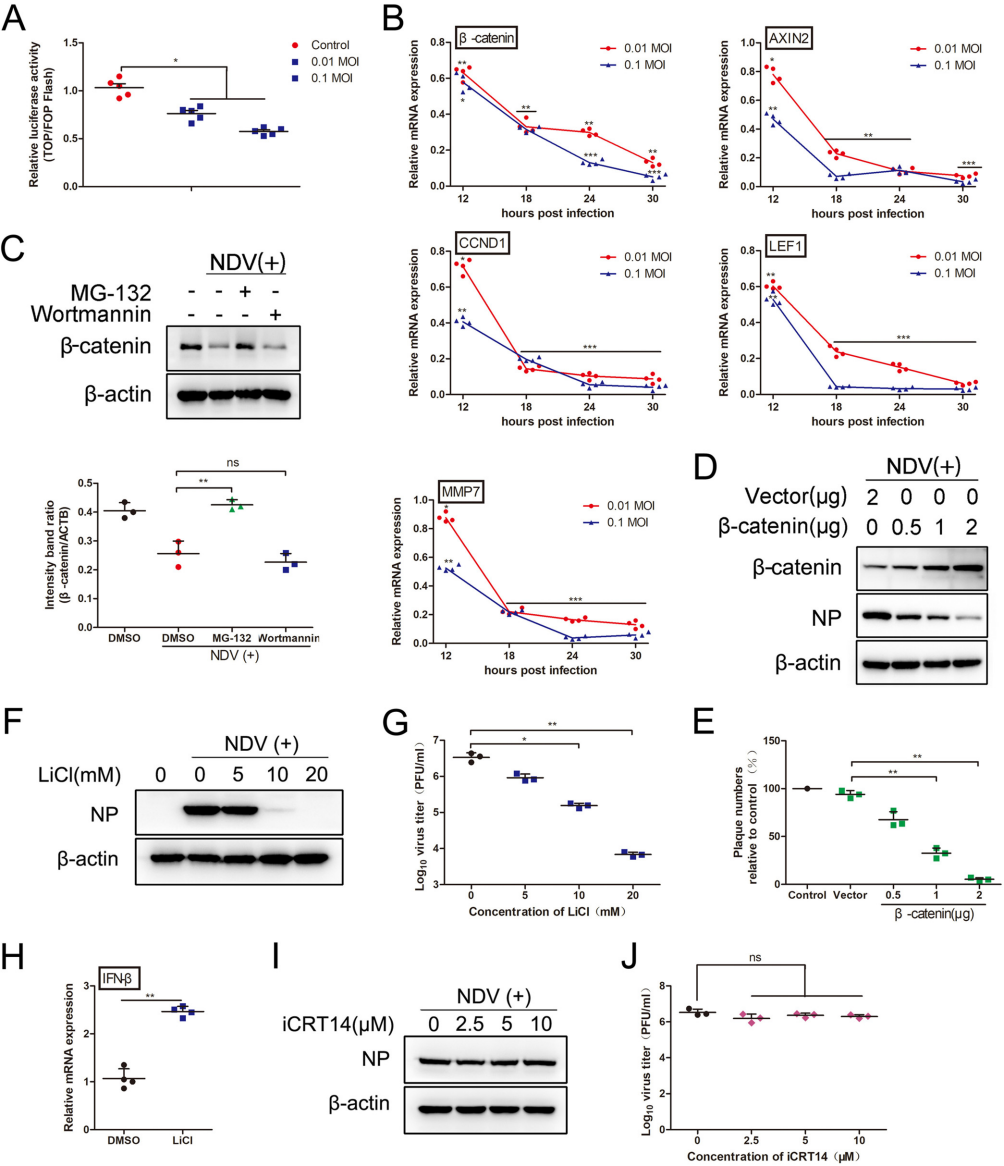
The Wnt/β-catenin signaling pathway regulates NDV proliferation. (A) NDV infection inhibits the activity of TOPFlash. DF-1 cells were transfected with TOPFlash or FOPFlash together with pRL-TK. At 24 h posttransfection, the cells were infected with F48E9 (0.01 or 0.1 MOI). At 24 h postinfection, the cells were harvested for the quantification of the luciferase activity. Mean values ± SDs are shown (*n* = 5). Significance was assessed via two-tailed, unpaired Student’s *t* tests. *, *P < *0.05. (B) NDV infection inhibits the downstream genes of the Wnt/β-catenin signaling pathway. DF-1 cells were infected with F48E9 (0.01 or 0.1 MOI). At 24 h postincubation, the cells were harvested to assess the expression of genes related to the β-catenin pathway. β-catenin, AXIN2, CCND1, LEF1, and MMP7. Mean values ± SDs are shown (*n* = 4). Significance was assessed via two-tailed, unpaired Student’s *t* tests. *, *P* < 0.05; **, *P < *0.01; ***, *P < *0.001. (C) NDV infection degrades the β-catenin through the ubiquitin-proteasome system. DF-1 cells were infected with F48E9 (0.01 MOI) and incubated with MG-132 (200 nM) or wortmannin (500 nM). At 24 h postincubation, the cells were harvested to assess the expression of protein via Western blotting (upper panel). The relative quantification of the target protein level was analyzed via ImageJ (lower panel). Mean values ± SDs are shown (*n* = 3). Significance was assessed via two-tailed, unpaired Student’s *t* tests. ns, not significant; **, *P < *0.01. (D and E) Exogenous expression of β-catenin inhibits NDV proliferation. DF-1 cells were transfected with pCAGGS-HA-β-catenin. At 24 h posttransfection, the cells were infected with F48E9 (0.01 MOI). At 24 h postinfection, the cells were harvested to assess the expression of viral protein via Western blotting (D). The supernatant was harvested to assess the viral titer via plaque assay (E). Mean values ± SDs are shown (*n* = 3). Significance was assessed via two-tailed, unpaired Student’s *t* tests. **, *P < *0.01. (F and G) LiCl inhibits NDV proliferation in DF-1 cells. DF-1 cells were infected with F48E9 (0.01 MOI) and incubated with LiCl. At 24 h postinfection, the cells were harvested to assess the expression of viral protein via Western blotting (F). The supernatant was harvested to assess the viral titer via plaque assay (G). Mean values ± SDs are shown (*n* = 3). Significance was assessed via two-tailed, unpaired Student’ s *t* tests. *, *P* < 0.05; **, *P < *0.01. (H) LiCl promotes the expression of IFN-β in DF-1 cells. DF-1 cells were incubated with LiCl (20 mM). At 24 h postincubation, the cells were harvested to assess the expression of IFN-β via qRT-PCR. Mean values ± SDs are shown (*n* = 4). Significance was assessed via two-tailed, unpaired Student’s *t* tests. **, *P < *0.01. (I and J) iCRT14 do not affect NDV proliferation in DF-1 cells. DF-1 cells were infected with F48E9 (0.01 MOI) and then incubated with iCRT14. At 24 h postinfection, the cells were harvested to assess the expression of viral protein via Western blotting (I). The supernatant was harvested to assess the viral titer via plaque assay (J). Mean values ± SDs are shown (*n* = 3). Significance was assessed via two-tailed, unpaired Student’s *t* tests. ns, not significant.

According to the transcriptome data, many genes downstream of the Wnt/β-catenin pathway were upregulated, including MYC (2.1287-fold), PPARgamma (2.7893-fold), and WISP1 (5.2201-fold), among others. The activation of the Wnt/β-catenin pathway is involved in viral infections, such as HSV and PRRSV, and β-catenin is the key effector protein (21, 22). Therefore, we investigated the relationship between the Wnt/β-catenin pathway and the proliferation of NDV. First, to clarify the effects of NDV infection on the Wnt/β-catenin pathway, DF-1 cells were infected with 0.01 or 0.1 MOI F48E9, and the TOPFlash/FOPFlash luciferase activity was determined. The TOPFlash reporter plasmid is a luciferase reporter of β-catenin-mediated transcriptional activation, and it contains seven TCF/LEF response elements. The activation of the Wnt/β-catenin pathway induces the binding of β-catenin to TCF/LEF and thereby activates the transcription of the Firefly luciferase reporter gene. The FOPFlash reporter plasmid is a control luciferase reporter that contains six mutated TCF/LEF binding sites (23). Using these reporter plasmids, we can detect β-catenin-mediated transcriptional activity. As shown in Fig. 5A, NDV infection reduced the luciferase activity in a dose-dependent manner. Consistent with this, the downstream genes of the Wnt pathway were significantly decreased upon F48E9 infection in a time-dependent manner (Fig. 5B). These results suggest that NDV infection dramatically inactivates the Wnt/β-catenin pathway. As shown in Fig. 5C, NDV infection caused the degradation of β-catenin, compared to the uninfected cells. Furthermore, MG-132 (an inhibitor of proteasome) treatment significantly restored the amounts of β-catenin, whereas wortmannin (an inhibitor of autophagy) had no effect on this. These results indicate that NDV infection leads to the degradation of β-catenin through the ubiquitin-proteasome system.

Second, to determine whether the β-catenin was involved in NDV infection, the β-catenin expression plasmid was constructed. As shown in Fig. 5D, the exogenous expression of β-catenin reduced the viral protein in a dose-dependent manner. When cells were transfected with β-catenin at a concentration of 1 or 2 μg, the viral titer was decreased to 29.94% or 4.50% of the control group, respectively (Fig. 5E). Similar results were observed when the Wnt/β-catenin pathway was stimulated by LiCl, which is an inhibitor of GSK-3β. The viral protein and viral titer were significantly decreased. LiCl (20 mM) caused a significant reduction of 2.69 Log in infectious virus particle production (Fig. 5F and G). β-catenin, as the key effector protein of the Wnt pathway, also plays an important role in the regulation of the immune response and pathogenic infection (13, 21). As expected, the expression of IFN-β was enhanced in the LiCl-treated cells (Fig. 5H). In contrast, the inhibition of the Wnt/β-catenin pathway by iCRT14 (iCRT14 inhibits β-catenin dependent transcription by interfering with the interactions of β-catenin interactions and TCF and by interfering with TCF binding to DNA) had no effect on the proliferation of NDV (Fig. 5I and J). These results demonstrate that the activation of the Wnt/β-catenin pathway suppresses NDV proliferation by enhancing the expression level of IFN-β.

**FIG 6:**
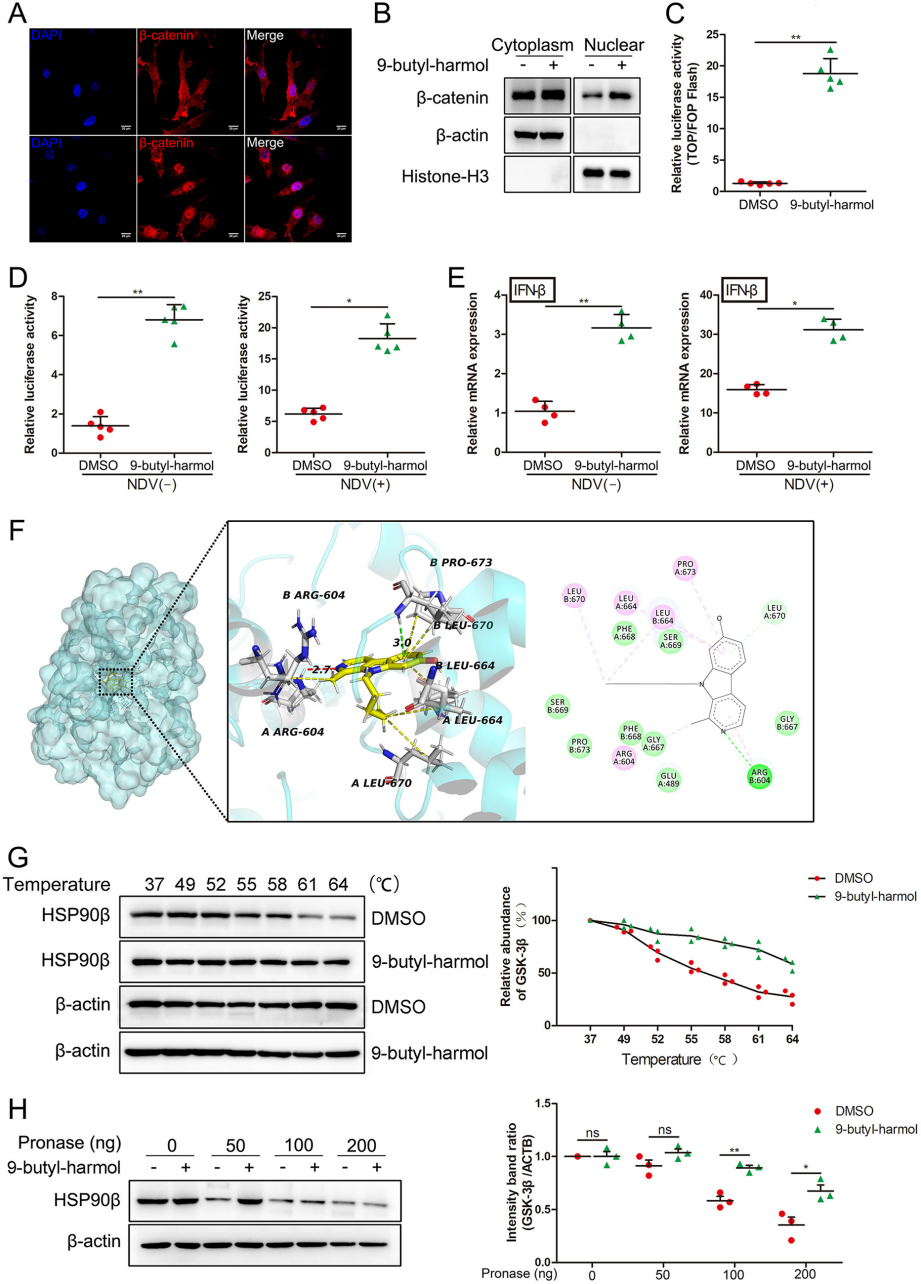
9-butyl-harmol enhanced the IFN-β-mediated immune response through the activation of the Wnt/β-catenin pathway, by targeting GSK-3β. (A and B) The accumulation of β-catenin in the nucleus of 9-butyl-harmol treated cells. DF-1 cells were treated with 9-butyl-harmol (5 μM). At 24 h posttreatment, the subcellular localization of β-catenin was detected via immunofluorescence microscopy (A) or Western blotting (B). (C) 9-butyl-harmol activates the activity of TOPFlash. DF-1 cells were transfected with TOPFlash or FOPFlash together with pRL-TK. At 24 h posttransfection, the cells were incubated with DMSO or 9-butyl-harmol (5 μM). At 24 h postincubation, the cells were harvested for the quantification of the luciferase activity (TOP/FOP). Mean values ± SDs are shown (*n* = 5). Significance was assessed via two-tailed, unpaired Student’s *t* tests. **, *P < *0.01. (D) 9-butyl-harmol promotes the chicken IFN-β promoter activity. DF-1 cells were transfected with chicken IFN-β-luc and pRL-TK. At 24 h posttransfection, the cells were infected with (right panel) or without (left panel) F48E9 (0.01 MOI) and then incubated with DMSO or 9-butyl-harmol (5 μM). At 24 h postincubation, the cells were harvested for the quantification of the luciferase activity. Mean values ± SDs are shown (*n* = 5). Significance was assessed via two-tailed, unpaired Student’s *t* tests. *, *P < *0.05; **, *P < *0.01. (E) 9-butyl-harmol promotes the expression of IFN-β. DF-1 cells were infected with (right panel) or without (left panel) F48E9 (0.01 MOI) and then incubated with DMSO or 9-butyl-harmol (5 μM). At 24 h postincubation, the cells were harvested to quantify the expression of IFN-β via qRT-PCR. Mean values ± SDs are shown (*n* = 4). Significance was assessed via two-tailed, unpaired Student’s *t* tests. *, *P < *0.05; **, *P < *0.01. (F) Molecular docking simulation of the binding of 9-butyl-harmol to GSK-3β. (G) 9-butyl-harmol protects GSK-3β from degradation due to heating. DF-1 cells were harvested and treated with DMSO or 9-butyl-harmol (200 μM). The thermal stability of GSK-3β was assessed via CETSA (left panel). The relative quantification of the target protein level was analyzed via ImageJ (right). (H) 9-butyl-harmol protects GSK-3β against proteolysis. DF-1 cells were harvested and treated with DMSO or 9-butyl-harmol (200 μM). The stability of GSK-3β against proteolysis was assessed via DARTS (left). The relative quantification of the target protein level was analyzed via ImageJ (right). Mean values ± SDs are shown (*n* = 3). Significance was assessed via two-tailed, unpaired Student’s *t* tests. ns, not significant; *, *P < *0.05; **, *P < *0.01.

To determine whether the Wnt/β-catenin pathway is activated by 9-butyl-harmol, the subcellular localization of β-catenin was analyzed. As expected, treatment with 9-butyl-harmol increased the accumulation of β-catenin in the nuclei (Fig. 6A and B). As shown in Fig. 6C, 9-butyl-harmol significantly activated the TOPFlash/FOPFlash luciferase activity. Overall, these results indicate that 9-butyl-harmol activates the Wnt/β-catenin pathway in DF-1 cells. The effect of 9-butyl-harmol on IFN-β was measured via qRT-PCR and luciferase activity. As shown in Fig. 6D, 9-butyl-harmol activated the IFN-β promoter activity with (2.89-fold) or without (4.90-fold) NDV infection. The expression of IFN-β was also upregulated with (1.95-fold) or without (3.04-fold) NDV infection (Fig. 6E). Besides, upon F48E9 infection, 9-butyl-harmol treatment increased the expression of OASL and MX1 to 2.51- and 3.13-fold, respectively (Fig. S5). These results indicate that 9-butyl-harmol activates the IFN-mediated immune response.

To predict whether 9-butyl-harmol has the potential to bind to GSK-3β, molecular docking was conducted. The interaction features were analyzed using Autodock 4 and Autodock Vina. The predicted inhibitory constant (*K_i_*) was 1.05 μM. The binding energy was predicted to be −8.16 kcal/mol. As shown in Fig. 6F, 9-butyl-harmol fits into the hydrophobic channel of the binding pocket and forms hydrophobic contacts with Val70, Ala83, Lys85, Met101, Val110, Leu132, and Phe201. The hydroxyl of 9-butyl-harmol formed two H bonds with Lys85 (1.66 Å) and Asp200 (1.89 Å). To further determine whether 9-butyl-harmol directly binds to GSK-3β, a cellular thermal shift assay (CETSA) and a drug affinity responsive target stability (DARTS) analysis were performed. CETSA is a drug-target identification method that is based on the principle that the engagement of a ligand to a protein could change the thermal stability of the target protein (24). As shown in Fig. 6G, 9-butyl-harmol strongly changed the thermal stability of GSK-3β at a variety of temperatures. DARTS is a label-free method that is based on the principle that the binding of a small molecule to a target protein could stabilize the target protein by increasing its resistance to proteases (25). Similar results were gained in DARTS. GSK-3β was less sensitive to proteolysis by pronase in the presence of 9-butyl-harmol (Fig. 6H). Collectively, our data suggest that 9-butyl-harmol directly binds to GSK-3β in DF-1 cells, which leads to the activation of immune responses that are mediated by IFN-β.

### 9-butyl-harmol destabilizes the NDV L protein by targeting HSP90β.

**FIG 7:**
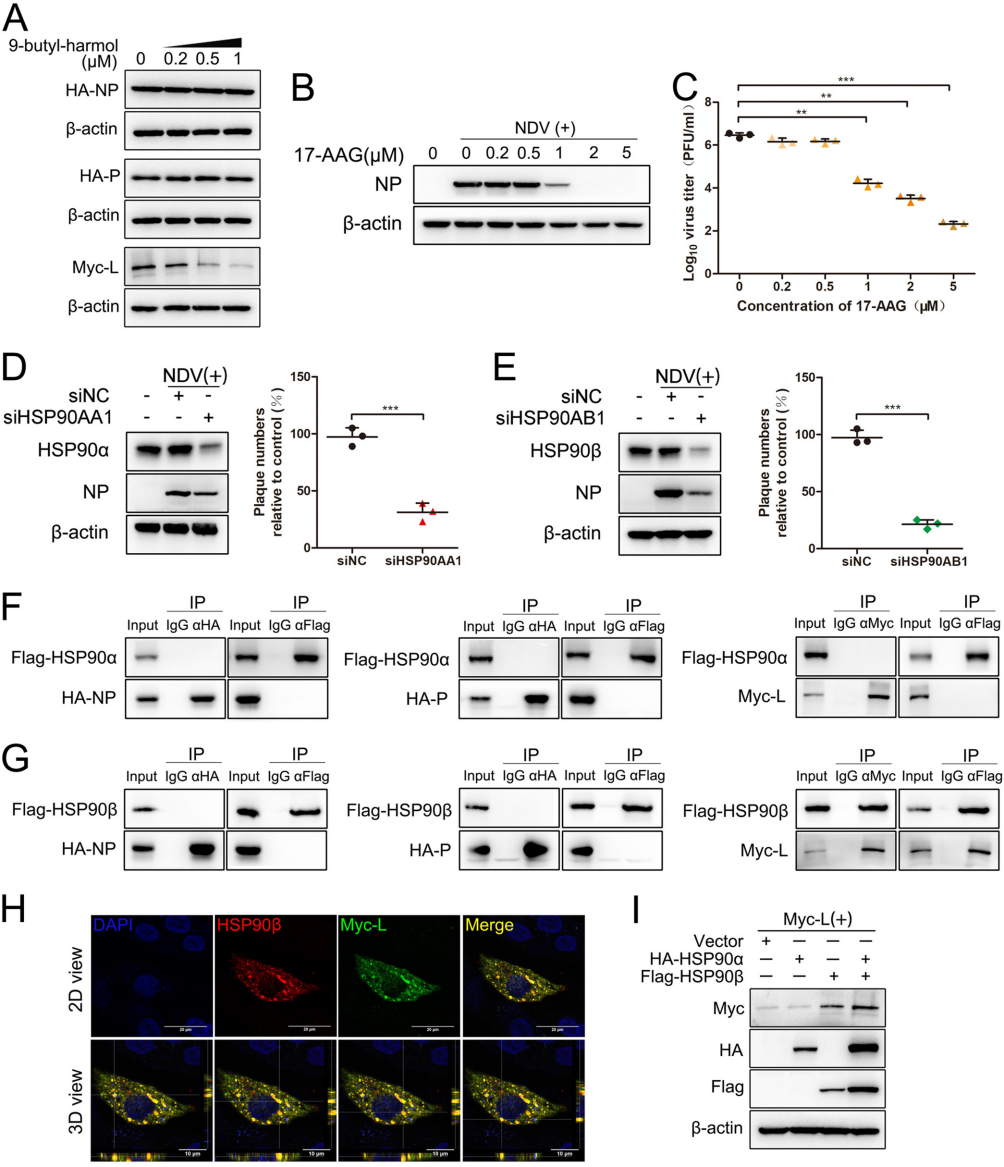
HSP90 facilitates NDV proliferation, and NDV L protein is a client protein of HSP90β. (A) 9-butyl-harmol suppresses the expression of the F48E9 L protein. DF-1 cells were transfected with plasmids encoding the NP, P, or L protein. At 6 h posttransfection, the medium was changed to fresh medium containing 9-butyl-harmol, and this was followed by 24 h of incubation. The cells were harvested to assess the expression of viral proteins via Western blotting. (B and C) 17-AAG inhibits NDV proliferation. DF-1 cells were infected with F48E9 (0.01 MOI) and incubated with 17-AAG. At 24 h postinfection, the cells were harvested to assess the expression of viral protein via Western blotting (B). The supernatant was harvested to assess the viral titer via plaque assay (C). Mean values ± SDs are shown (*n* = 3). Significance was assessed via two-tailed, unpaired Student’s *t* tests. **, *P < *0.01; ***, *P < *0.001. (D) The knockdown of HSP90α inhibits NDV proliferation. DF-1 cells were transfected with siHSP90AA1-1. At 48 h posttransfection, the cells were infected with F48E9 (0.01 MOI). At 24 h postinfection, the cells were harvested to assess the expression of viral protein via Western blotting (left panel). The supernatant was harvested to assess the viral titer via plaque assay (right panel). Mean values ± SDs are shown (*n* = 3). Significance was assessed via two-tailed, unpaired Student’s *t* tests. **, *P < *0.01. (E) The knockdown of HSP90β inhibits NDV proliferation. DF-1 cells were transfected with siHSP90AB1-2. At 48 h posttransfection, the cells were infected with F48E9 (0.01 MOI). At 24 h postinfection, the cells were harvested to assess the expression of viral protein via Western blotting (E). The supernatant was harvested to assess the viral titer via plaque assay (F). Mean values ± SDs are shown (*n* = 3). Significance was assessed via two-tailed, unpaired Student’s *t* tests. ***, *P < *0.001. (F) NDV viral proteins do not interact with HSP90α. DF-1 cells were transfected with plasmids of viral proteins (NP, P, or L) together with pCAGGS-Flag-HSP90AA1. At 48 h posttransfection, the interaction between viral proteins and HSP90α was analyzed via coimmunoprecipitation assay. (G) NDV L protein interacts with HSP90β. DF-1 cells were transfected with plasmids of viral proteins (NP, P, or L) together with pCAGGS-Flag-HSP90AB1. At 48 h posttransfection, the interaction between viral proteins and HSP90β was analyzed via coimmunoprecipitation assay. (H) Colocalization of HSP90β and the NDV L protein. DF-1 cells were transfected with pCAGGS-myc-L together with pCAGGS-Flag-HSP90AB1. At 48 h posttransfection, the subcellular localization of two proteins was analyzed via immunofluorescence microscopy. (I) HSP90β enhances the expression level of the NDV L protein. DF-1 cells were transfected with pCAGGS-Flag-HSP90AA1, pCAGGS-Flag-HSP90AB1, or both plasmids together with pCAGGS-myc-L. At 48 h posttransfection, the expression of the protein was analyzed via Western blotting.

According to the GO analysis, many DEGs are involved in protein folding, leading us to question whether 9-butyl-harmol affects the viral proteins to modulate NDV proliferation. The RNA synthesis of NDV relies on the ribonucleoprotein (RNP) complex, which is composed of the NP, P, L, and RNA genome (26). To test the hypothesis that 9-butyl-harmol might affect protein folding or stability, DF-1 cells were transfected with plasmids encoding viral proteins of the RNP complex and were treated with either 9-butyl-harmol or DMSO. The results showed that treatment with 9-butyl-harmol led to a significant reduction of the expression of L. In the meantime, the expression of NP and the expression of P were not affected (Fig. 7A). One of the DEGs that is involved in protein folding is HSP90. HSP90 plays a crucial role in protein folding and maturation (27). Viral proteins also rely on HSP90 to mediate their folding and stabilization (15). To determine the relationship between HSP90 and NDV, the following experiments were conducted. The effect of NDV infection on HSP90 expression was determined via Western blotting. As shown in Fig. S6, NDV infection had no effect on the expression of HSP90α, regardless of the MOI of NDV. Interestingly, high doses of NDV (1 MOI) slightly enhanced the amounts of HSP90β, whereas low doses of NDV (0.01 or 0.1 MOI) had no effect. 17-AAG, an inhibitor of HSP90, significantly reduced the viral protein in a dose-dependent manner (Fig. 7B). In the presence of 5 μM 17-AAG, infectious virus particle production was reduced by 4.19 Log, compared to cells treated with DMSO (Fig. 7C). To further confirm whether HSP90 plays a role in NDV infection, siRNAs targeting HSP90AA1 or HSP90AB1 were synthesized, and their interference efficiencies were detected via qRT-PCR. As shown in Fig. S7A and S7B, siHSP90AA1-1 and siHSP90AB1-2 were used for follow-up testing due to their interference efficiency. In the absence of HSP90AA1 or HSP90AB1, NDV proliferation was markedly reduced by 68.67% and 78.71%, respectively (Fig. 7D and E). These results suggest that the inhibition of HSP90 significantly suppresses NDV proliferation.

To further explore the underlying mechanism, plasmids encoding HSP90AA1 and HSP90AB1 were constructed. The interactions between HSP90 and the NP, P, and L proteins were analyzed via coimmunoprecipitation (Co-IP). As illustrated in Fig. 7F, HSP90α was not coimmunoprecipitated with any of the viral proteins of the RNP complex. Only the L protein was coimmunoprecipitated with HSP90β (Fig. 7G). The localization of the L protein and HSP90β were further examined via immunofluorescence using a confocal microscope. As shown in Fig. 7H, when L and HSP90β were coexpressed, the localization of the two proteins was highly consistent. As shown in Fig. 7I, when HSP90β and L were coexpressed, the amounts of L protein were markedly increased, whereas HSP90α had no influence. Interestingly, when HSP90α, HSP90β, and L were coexpressed, the amounts of these proteins were all increased. These results demonstrate that the NDV L protein is a client protein of HSP90β.

**FIG 8:**
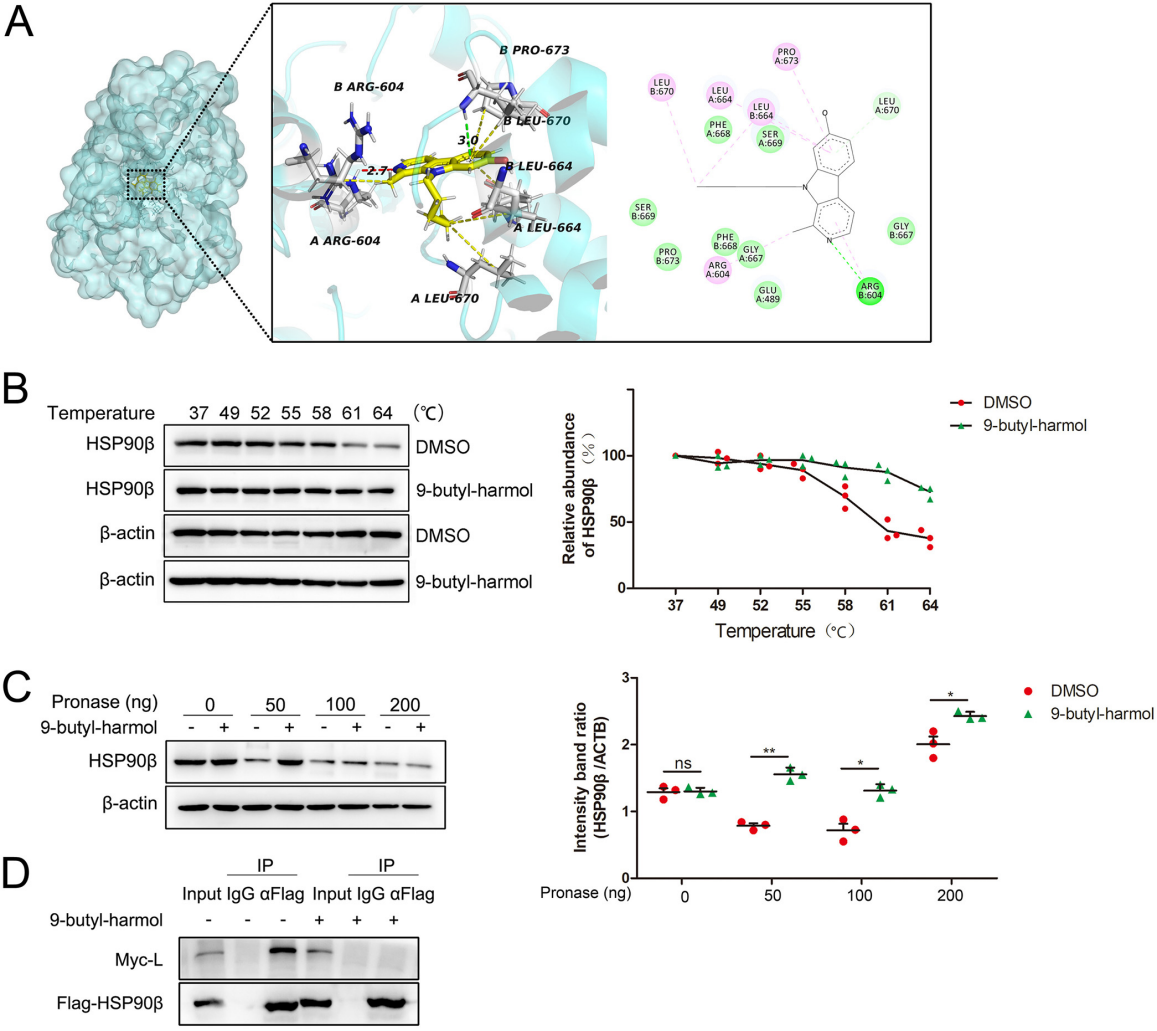
Validation of HSP90β as a target of 9-butyl-harmol. (A) Molecular docking simulation of the binding of 9-butyl-harmol to HSP90β. (B) 9-butyl-harmol protects HSP90β from degradation due to heating. DF-1 cells were harvested and treated with DMSO or 9-butyl-harmol (200 μM). The thermal stability of HSP90β was assessed via CETSA (left panel). The relative quantification of the target protein level was analyzed via ImageJ (right panel). (C) 9-butyl-harmol protects HSP90β against proteolysis. DF-1 cells were harvested and treated with DMSO or 9-butyl-harmol (200 μM). The stability of HSP90β against proteolysis was assessed via DARTS (left panel). The relative quantification of the target protein level was analyzed via ImageJ (right). Mean values ± SDs are shown (*n* = 3). Significance was assessed via two-tailed, unpaired Student’s *t* tests. ns, not significant; *, *P < *0.05; **, *P < *0.01. (D) 9-butyl-harmol disrupts the interaction between HSP90β and the NDV L protein. DF-1 cells were transfected with pCAGGS-myc-L together with pCAGGS-Flag-HSP90AB1. At 42 h posttransfection, the cells were treated with or without 9-butyl-harmol (10 μM), and this was followed by 6 h of incubation. The cells were then harvested and immunoprecipitated with the Flag antibody, with or without 9-butyl-harmol (20 μM).

Previous studies have shown that several β-carboline compounds could bind to HSP90 (28, 29). To predict whether 9-butyl-harmol has the potential to bind to HSP90, molecular docking was conducted. As shown in Fig. 8A, 9-butyl-harmol fits perfectly into the binding pocket of HSP90 (PDB ID: 5FWK). The predicted inhibitory constant (*K_i_*) was 558.06 nM. The binding energy was predicted to be −8.53 kcal/mol. 9-butyl-harmol was predicted to form a pi-alkyl interaction and a weak H bond with Arg604. Three pi-alkyl interactions were formed between Leu664 and the A ring of 9-butyl-harmol. In addition, hydrophobic contacts were formed with Leu670 and Pro673. To further validate the binding of 9-butyl-harmol to HSP90β, CETSA and DARTS were performed. As shown in Fig. 8B, compared to the vehicle control, 9-butyl-harmol increased the thermal stability of cellular HSP90β. β-actin, as a control protein, was not protected by the 9-butyl-harmol treatment. The results of the DARTS analysis showed that the pronase-induced digestion of HSP90β was prevented by 9-butyl-harmol with 50 ng of pronase, and the amounts of β-actin were unchanged by 9-butyl-harmol treatment (Fig. 8C). Next, we investigated whether 9-butyl-harmol affected the formation of the HSP90β-L complex. After the 9-butyl-harmol treatment, the amount of L protein was dramatically decreased. The results suggested that 9-butyl-harmol destroyed the HSP90β-L interaction (Fig. 8D). Taken together, our data suggest that 9-butyl-harmol directly interacts with HSP90β and disrupts the HSP90β-L interaction, thereby resulting in the inhibition of NDV proliferation.

**FIG 9:**
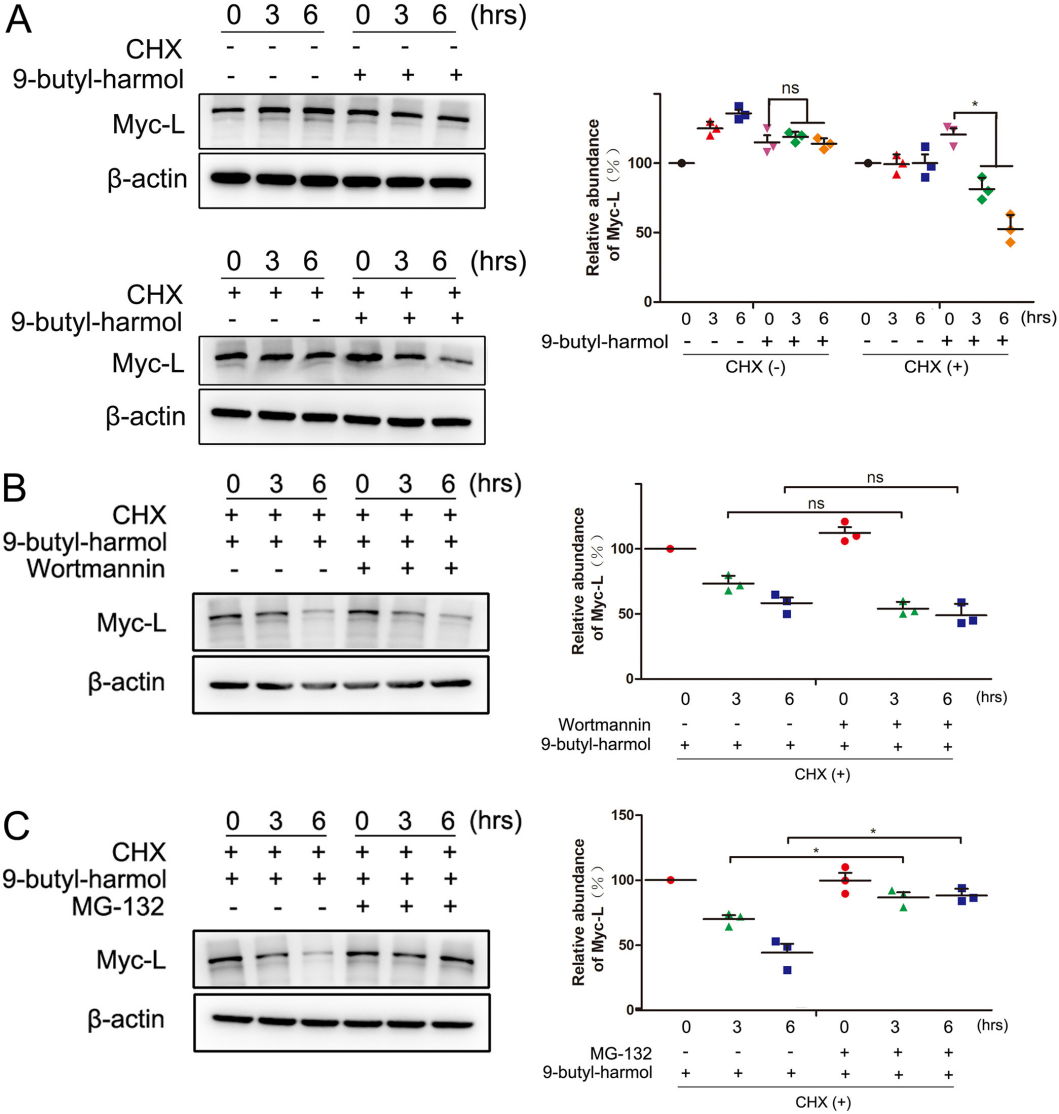
9-butyl-harmol induces the degradation of the NDV L protein through the ubiquitin-proteasome pathway. (A) 9-butyl-harmol induces the degradation of the NDV L protein. DF-1 cells were transfected with pCAGGS-myc-L. At 48 h posttransfection, the cells were treated with (lower left panel) or without (upper left panel) cycloheximide (50 μg/mL) for 30 min, after which the cells were incubated with DMSO or 9-butyl-harmol (5 μM). At the indicated time points, the cells were harvested to assess the expression level of the L protein via Western blotting. The relative quantification of the target protein level was analyzed via ImageJ (right panel). Mean values ± SDs are shown (*n* = 3). Significance was assessed via two-tailed, unpaired Student’s *t* tests. ns, not significant, *, *P < *0.05. (B) NDV L protein degradation does not rely on autophagy. DF-1 cells were transfected with pCAGGS-myc-L. At 48 h posttransfection, the cells were treated with cycloheximide (50 μg/mL) for 30 min, after which the cells were incubated with 9-butyl-harmol (5 μM) together with wortmannin (500 nM). At the indicated time points, the cells were harvested to assess the expression level of the L protein via Western blotting (left panel). The relative quantification of the target protein level was analyzed by ImageJ (right panel). Mean values ± SDs are shown (*n* = 3). Significance was assessed via two-tailed, unpaired Student’s *t* tests. ns, not significant. (C) The NDV L protein is degraded through the ubiquitin-proteasome pathway. DF-1 cells were transfected with pCAGGS-myc-L. At 48 h posttransfection, the cells were treated with cycloheximide (50 μg/mL) for 30 min, after which the cells were incubated with 9-butyl-harmol (5 μM) together with MG-132 (200 nM). At the indicated time points, the cells were harvested to assess the expression level of the L protein (left). The relative quantification of the target protein level was analyzed via ImageJ via Western blotting (right panel). Mean values ± SDs are shown (*n* = 3). Significance was assessed via two-tailed, unpaired Student’s *t* tests. *, *P < *0.05.

Many HSP90 client proteins are destabilized when the HSP90 activity is interrupted (30). To determine whether the L protein was degraded upon 9-butyl-harmol treatment, the effect of 9-butyl-harmol on the L protein was analyzed in the presence of cycloheximide (CHX), which is a protein synthesis inhibitor. Under the condition in which cells were treated with CHX, the amounts of L protein were markedly reduced at 6 h posttreatment, indicating that the stability of the L protein was changed under the 9-butyl-harmol treatment (Fig. 9A). To further explore the mechanism of the degradation of the L protein that was caused by 9-butyl-harmol treatment, 9-butyl-harmol-treated cells were treated with wortmannin or MG-132. As shown in Fig. 9B, in the presence of wortmannin, no change in protein levels was observed. However, in the combined presence of MG-132 and 9-butyl-harmol, the amounts of L protein were restored, showing a significant recovery from the 9-butyl-harmol-only treatment group (Fig. 9C). These data confirm a role for 9-butyl-harmol in the destabilization of the L protein and prove that the L protein is degraded via the ubiquitin-proteasome system.

**FIG 10:**
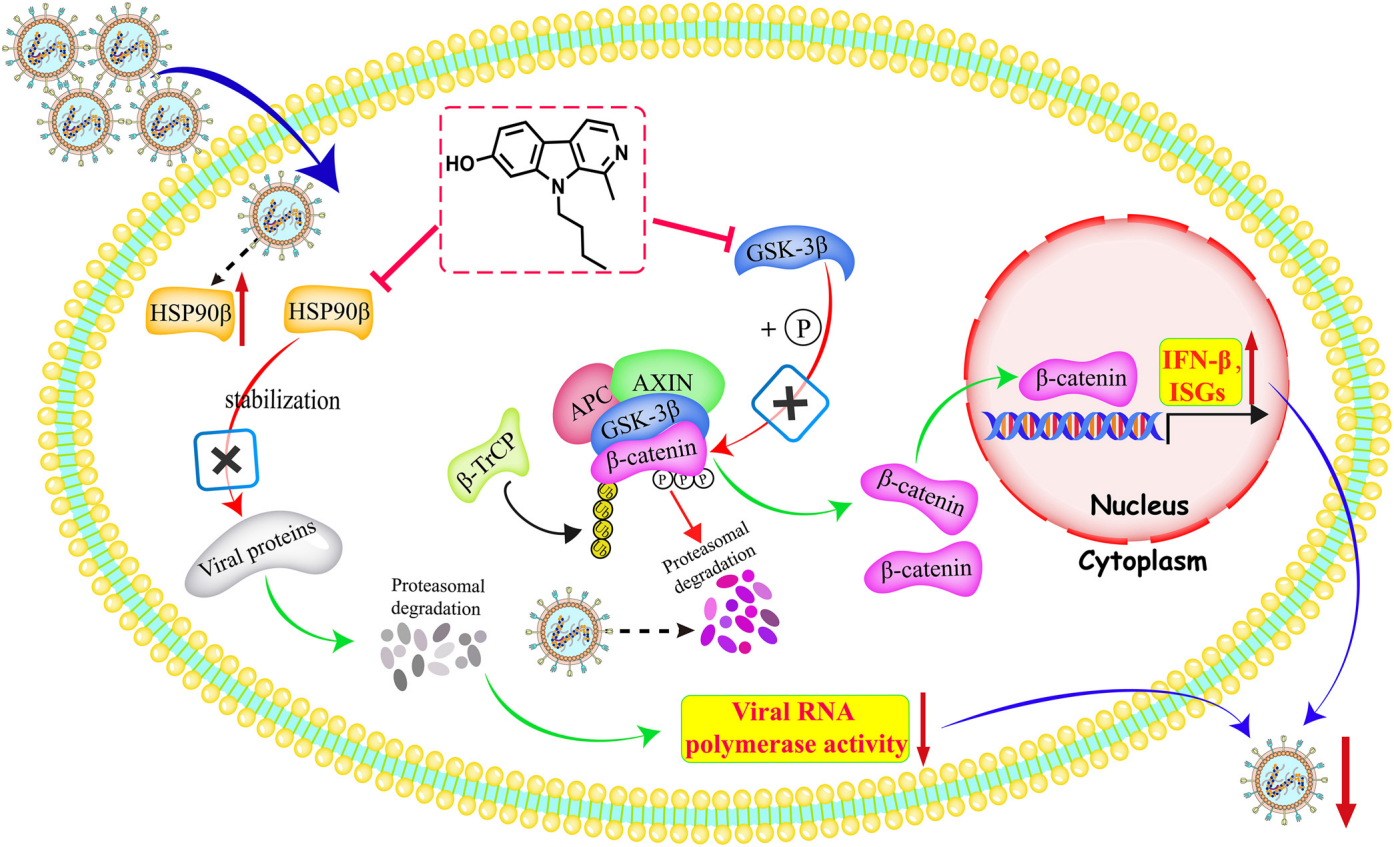
Illustration of the mechanism underlying the antiviral effect of 9-butyl-harmol against NDV.

Overall, our findings reveal the antiviral mechanism of 9-butyl-harmol against NDV (Fig. 10).

## DISCUSSION

β-carboline alkaloids have various pharmacological activities, including antiviral, antidiabetic, neuroprotective, antitumor, and anti-inflammatory. Harmine, isolated from Peganum harmala L., is a well-studied β-carboline alkaloid. It exerts its biological functions through multiple targets. Harmine exerts its neuroprotective and antidiabetic effect through the inhibition of DYRK1A. It also can ameliorate memory impairment through binding acetylcholine esterase (AChE). Monoamine oxidase A (MAO-A) can serve as another target of harmine, which contributes to its antidepressant effect (31). However, the antiviral target of β-carboline alkaloids has seldom been researched. To date, most studies about the antiviral activity of β-carboline alkaloids focused on biological activity and structural modification. Here, we confirmed GSK-3β, which leads to the regulation of the Wnt pathway, as the target of 9-butyl-harmol. Manzamine A and related derivatives, a subclass of the β-carboline alkaloids, were proven to inhibit GSK-3β (32). GNF7156 was reported to induce human β-cell proliferation through the inhibition of GSK-3β and DYRK1A, indicating that the three-dimensional structures of their binding pockets might be similar (33). Meanwhile, harmine is an inhibitor of DYRK1A (31). This literature indicated that β-carboline alkaloids have the potential to interact with GSK-3β. Recently, harmine derivatives were identified as GSK-3β/DYRK1A dual inhibitors, thereby serving as potent candidates for Alzheimer’s disease (17). Our data proved that 9-butyl-harmol dramatically increased the stability of GSK-3β against heating and pronase treatment, demonstrating that 9-butyl-harmol could directly bind to GSK-3β. These results provide more evidence that β-carboline alkaloids could serve as novel inhibitors of GSK-3β. Furthermore, due to the biological activity of GSK-3β, we speculate that 9-butyl-harmol also has the potential to serve as a candidate for diabetes mellitus and Alzheimer’s disease.

β-catenin, as the key effector protein of the Wnt pathway, activates the IFN-β pathway and induces the production of various ISGs during both DNA and RNA virus infection. It enhances the virus-induced production of IFN-β by binding to the C-terminal domain of IRF3 and recruiting CBP/p300 (12). In addition, β-catenin upregulates the expression of IFN-β through the TCF binding sites that are present on the IFN-β promoter, independent of IRF3 (13). Accordingly, viruses have evolved corresponding strategies by which to combat the β-catenin-mediated immune response. The US3 protein of HSV-1 targets β-catenin to inhibit cGAS/STING-mediated IFN-β production for the purpose of evading the cellular immune response (22). The influenza A virus suppresses β-catenin-dependent IFN-β production via the activation of the RIG-I/NF-κB signaling cascade (18). However, the role of β-catenin in NDV infection remains unknown. In this study, we demonstrated that the suppression of GSK-3β led to the inhibition of NDV by enhancing the expression of IFN-β. NDV infection inactivated the Wnt pathway and significantly degraded β-catenin via the ubiquitin-proteasome system. Furthermore, we attempted to investigate the mechanism. Seven plasmids that express viral genes (NP, P, L, M, HN, F, and V) were transfected into DF-1 cells, and none of these proteins had an effect on the degradation of β-catenin (data not shown). However, upon human cytomegalovirus infection, viral proteins were proven to inhibit β-catenin (19). Thus, the mechanism underlying the inactivation of the Wnt pathway by NDV remains to be studied.

HSP90β was identified as another target of 9-butyl-harmol and contributed to the folding and stability of the L protein. HSP90 is a highly abundant molecular chaperone that plays a pivotal role in the maintenance of cellular homeostasis under changing conditions. To date, HSP90 seems to be an attractive target for antiviral agents, as the effective proliferation of many viruses relies upon HSP90. On one hand, viral proteins are synthesized and accumulated in large quantities in a short period, placing a heavy burden upon the cellular protein folding machinery. On the other hand, the structures of viral proteins are complex, which leads to a susceptibility to misfolding. For these reasons, viral proliferation shows hypersensitivity to HSP90 inhibition at concentrations that do not harm cellular viability (15, 20, 34). There are two isoforms that are located in the cytoplasm: HSP90α and HSP90β. HSP90α is considered to be one stress-inducible chaperone, whereas HSP90β is a constitutively expressed protein (35). They facilitate the folding of *de novo* synthesized and incorrectly folded proteins (36). Several reports found that HSP90 is essential to the folding of the L protein of the respiratory syncytial virus, measles virus, and Nipah Virus (37, 38). However, these studies did not distinguish which specific protein was responsible for the folding of L. Here, we found that the NDV L protein is a client of HSP90β but not of HSP90α. The approximately 250 kDa L of paramyxoviruses is a remarkable enzyme that both transcribes and replicates the entire genome. The formation of HSP90β-L complexes enhanced the stability of the L protein, thereby preventing the L protein from degradation. Six conserved domains were identified from other nonsegmented, negative-strand viruses, RNA-dependent RNA polymerase (RdRp), poly-ribonucleotidyltransferase (PRNTase), connecting domain (CD), methyltransferase (MTase), and the C-terminal domain (CTD) (39). The complex structure of L leads to a susceptibility to misfolding and a lack of stability. The previous report found that harmine directly binds Plasmodium falciparum HSP90 to exert its anti-malarial activity. Interestingly, harmalol binds human HSP90 more tightly than Plasmodium falciparum HSP90, indicating that the hydroxyl substituent at C^7^ was responsible for this affinity transition (16). Consistent with these data, we found that the antiviral activity of 9-butyl-harmol was better than that of 9-butyl-harmine. The underlying mechanism might be that 9-butyl-harmol binds HSP90β more tightly than does 9-butyl-harmine. Meanwhile, harmine cannot bind human HSP90 (16), which explains why harmine had no effect on NDV proliferation in different cell lines. These data also illustrate that the butyl substituent at N^9^ is essential for the interaction between HSP90β and 9-butyl-harmol.

However, HSP90α and HSP90β are highly homologous, with an 85% consistent sequence (40). Thus, most HSP90 inhibitors have inhibitory activity against both HSP90α and HSP90β. We assume that 9-butyl-harmine is no exception. Our data suggest that the knockdown of HSP90α also inhibited NDV proliferation and that NP, P, and L are not client proteins of HSP90α. However, our results showed that HSP90α could facilitate the expression of HSP90β, which may be the reason for the antiviral effect of the knockdown of HSP90α. We cannot rule out the possibility that other viral proteins may be the client protein of HSP90α or HSP90β. Thus, we will further investigate this possibility. In this study, we presented two independent antiviral mechanisms that target GSK-3β and HSP90. However, we suggest that the HSP90-related mechanism plays a major role in the anti-NDV activity of 9-butyl-harmol, based on two results: (i) the effects of IFN induction and viral protein degradation that are induced by 9-butyl-harmol and (ii) the antiviral effects of the activation of β-catenin signaling (LiCl) and the inhibition of HSP90 (17-AAG).

Harmine was reported to inhibit HSV proliferation. However, our data showed that harmine has no effect on NDV proliferation. Thus, we further tested the antiviral activity of harmine against PRV, another member of Herpesviridae. Interestingly, harmine exhibited excellent anti-PRV activity (data unpublished). These data suggest that β-carboline compounds exhibit different antiviral mechanisms against DNA and RNA viruses and that the target of harmine has no effect on RNA virus proliferation. In contrast, 9-butyl-harmol inhibits a wide range of viruses, indicating that its antiviral mechanism is not strain-specific. Furthermore, 9-butyl-harmol has a stronger inhibitory effect on RNA viruses than on DNA viruses. This is a sign that the targets of 9-butyl-harmol play more important roles in the proliferation of RNA viruses than in the proliferation of DNA viruses. It has more potential to serve as an antiviral agent against RNA viruses, especially paramyxovirus.

In this study, we screened a series of β-carboline derivatives for antiviral agents against NDV. 9-butyl-harmol was identified as an excellent antiviral agent against a broad range of viruses, especially paramyxoviruses. The study of the mode of action demonstrated that 9-butyl-harmol exerts its antiviral activity in the postentry phase. We further elucidated the antiviral mechanism of 9-butyl-harmol and provided important virologic insights into the proliferation of NDV. These data also provide potential targets for the development of antiviral agents against NDV and other paramyxoviruses.

## MATERIALS AND METHODS

### Cells and viruses.

The cell lines DF-1 (ATCC CRL-12203), BHK-21 (CCL-10), Vero (CCL-81), MARC-145 (CRL-12231), HeLa (CCL-2), and A549 (CCL-185) were routinely maintained in Dulbecco’s modified Eagle’s medium (DMEM, Gibco) supplemented with 10% vol/vol fetal bovine serum (FBS, SeraPro) at 37°C with 5% CO_2_ in an incubator (Thermo). F48E9, La Sota, PPMV-1/SX-01/Ch/15 (SX01), Blackbird/China/08, PPRV Nigeria 75/1, PRRSV, AIV, VSV, and PRV were provided by the College of Veterinary Medicine, Northwest A&F University (Yangling, China). CDV-11 was purchased from Qilu Animal Health (China).

### Chemicals and antibodies.

Wortmannin (catalog number T6283), iCRT14 (catalog number T4486), 17-AAG (catalog number T6290), protease inhibitor cocktail (catalog number C0001), and phosphatase inhibitor cocktail I (catalog number C0002) were purchased from TargetMol (USA). Cycloheximide (catalog number S7418) was purchased from Selleck (USA). MG-132 (catalog number HY-13259) was purchased from MedChemExpress (USA). LiCl (catalog number L9650) and Pronase (catalog number P8811) were purchased from Sigma-Aldrich (USA). The β-carboline derivatives were synthesized as previously described (41, 42).

GSK-3β (D5C5Z) XP Rabbit MAb (catalog number 12456), HA-Tag (C29F4) Rabbit MAb (catalog number 3724), DYKDDDDK Tag (9A3) mouse MAb (catalog number 8146), Anti-rabbit IgG, HRP-linked Antibody (catalog number 7074), and Anti-mouse IgG, HRP-linked Antibody (catalog number 7076) were purchased from Cell Signaling Technology (USA). HSP90 Polyclonal Antibody (catalog number 13171-1-AP), HSP90 Monoclonal Antibody (catalog number 60318-1-Ig), HSP90AB1 Polyclonal Antibody (catalog number 11405-1-AP), Beta Catenin Polyclonal Antibody (catalog number 51067-2-AP), Beta Actin Monoclonal Antibody (catalog number 66009-1-Ig), MYC-Tag antibody (catalog number 16286-1-AP), and Histone-H3 Polyclonal Antibody (catalog number 17168-1-AP) were purchased from Proteintech (USA). NDV NP Monoclonal Antibody was prepared in our laboratory. Alexa Fluor 594 AffiniPure Goat Anti-Rabbit IgG (H+L) (catalog number 33112ES60), Alexa Fluor 594 AffiniPure Goat Anti-Mouse IgG (H+L) (catalog number 33212ES60), and Alexa Fluor 488 AffiniPure Goat Anti-Mouse IgG (H+L) (catalog number 33206ES60) were purchased from Yeasen (China).

### Cell viability assay.

The cell viability assay was performed using the CCK-8 reagent (catalog number C0005) (TargetMol, USA) as previously described (8). Briefly, cells were dispersed into 96-well plates and cultured for 24 h before different concentrations of compounds were added to each well. At 48 h postincubation, 10 μL of the CCK-8 reagent were added to each well, and the plates were incubated for 2 h. Then, the optical density was measured at 450 nm.

### Plaque assay.

The plaque assay was performed as previously described (8). BHK-21 cells were seeded in 24-well plates and were infected with a series of diluted samples. After adsorption at 37°C for 1 h, unbound virions were removed via washing with phosphate-buffered saline (PBS). DF-1 cells were covered with medium containing methyl cellulose (1%). The cells were fixed and stained with crystal violet solution for 30 min. Plaques were visualized, and the virus titer was calculated.

### RNA isolation and qRT-PCR analysis.

RNA isolation and quantitative real-time PCR (qRT-PCR) were performed as previously described (8). RNA was extracted using RNAiso Plus (TaKaRa), according to the manufacturer’s instructions. qRT-PCR was performed using 2 × RealStar green power mixture (catalog number A301-01) (GenStar, China), according to the manufacturer’s instructions. The relative expressions of the target gene levels were calculated by using the 2^-ΔΔCT^ method as previously described (43). The primers that were used in this study are listed in Table 4.

**TABLE 4:** The sequences of the primers used in this study

Gene name	Forward primer (5′–3′)	Reverse primer (5′–3′)
β-catenin	ATGGCAACCCAAGCTGACTTGATG	TTACAGGTCAGTATCGAACCAGGC
HSP90AA1	ATGCCGGAAGCTGTGCAAA	TTAATCCACCTCCTCCATACGTGA
HSP90AB1	ATGCCCGAGCAAGTGCAGCATGGA	TTAGTCCACCTCTTCCATGCGGGA
q-β-catenin	TTATTCTGGCAAGCGGTGGAC	TTCAGCACCCTACTTGTGGTC
q-HSP90AA1	AAACCAGAGATTGAGGACGTT	TCCCCGTACTCCTCATTGGTG
q-HSP90AB1	AAATTGAGGATGTGGGCTCT	CTTGTTCAGCTCCTCCTGGTC
q-CCND1	TGAAATGGAATCTGGCTGCAA	AGCTGCGATCATGGAAGGTG
q-MMP7	CAAAAGAGTTACCTCGGGACA	GCCAAATTCATGAGCAGCAAC
q-LEF1	TTTCCCCAGGCTCCCACCCAT	AGCTCACTGTCGTTGTGCGGAT
q-Axin2	TCCAGTGATGCCCTGACGGAT	TGACCGTTGGCCTTAACACTGC
q-GHR	TTGGACATAGATGACTCCGAT	GGTTCATAACAACTGGCACGTC
q-HSPB1	GCGCCGACAGCTGGAAGGTCA	TGCTCATCCTGTTTCTCCTCGT
q-CXCL12	CTGACTTACCGATGCCCCTGT	GCTTGGGATCAATGCACACT
q-SMAD3	CAACCAGGAATTCGCAGCTCT	TAGTCACCGTCTGCCGCCTGT
q-TGFβ3	GACACCGTGCGTGAGTGGCTT	TGTTGGGCTCTCCAGGCGATG
q-IFITM3	CGGCCCATCTGATCAACGTCT	GGTCCAATGAATTCGGGGTGT
q-MYC	CACGCCGCCCACGACCAGCAG	TGGCGGCTGGGTATTCCACCT
q-TMEM26	GTTCAGTTAAATGTCACCGAT	CCCGTGTGATTTCAACCCCAA
q-IRF1	TAAAGTAGGCGAGAAGGACCC	AGCATCCTGTACACTCGGACA
q-IRF7	GCCGTATCTTCCGCATCCCT	TCAGCGTTCCCCTCGTACCTG
q-CCL4	CCAGAGGCACTACAGCACCAG	TCAGTCCCGCTTGACGCTCT
q-IFN-β	TTCTCCTGCAACCATCTTCGT	TGGCTGCTTGCTTCTTGTCC
q-MX1	AAGCCTGAGCATGAGCAGAA	TCTCAGGCTGTCAACAAGATCAA
q-OASL	AGATGTTGAAGCCGAAGTACCC	CTGAAGTCCTCCCTGCCTGT
q-PRV	AGAGCGACAACGAGACGCCCAG	GCGGACAGCGAGCAGATGACCAG
q-CDV	TCTTTGAGGCGATTCATGGTG	AAACTCATGCAACCCAAGAGC
q-PPRV	CATTACCCGTTCAAGACTGCT	AACTACCTCAACAAGGCGGAT
q-F48E9(P)	TCATGCCCAGCTACCTGTCG	CTGTTGGATTTCAGACCGCATC
q-F48E9(NP)	TGCGGTATTCTGCCTTCGGAT	GCACGCTGTTGGTAAAGCCAT
q-PRRSV	ATCATCGCCCAACAAAACCAG	CGTCGGCAAACTAAACTCCAC
q-VSV	TACCCGGACCACATGAAGCAG	GCTTGTGGCCGAGGATGTTGC
q-AIV	GGCAGGGAATGGTAGATGGT	GGTGACTCCGTCTATTGCCTT
28S rRNA(DF-1)	GGTATGGGCCCGACGCT	CCGATGCCGACGCTCAT
q-GAPDH(Vero and MARC-145)	TCCGACTTCAACAGCGACACC	TGTTGCTGTAGCCAAATTCGT
q-GAPDH(BHK-21)	AACCTGCCAAGTATGAGGACA	ATGTAGGCCATGAGGTCCACCA
q-GAPDH(A549)	TCTTCCAGGAGCGAGATCCCT	AGCAGTTGGTGGTGCAGGAGG

### Adsorption assay.

Adsorption assays were performed as reported previously, with some modifications (44). Briefly, DF-1 cells were treated as described below. (i) DF-1 cells were prechilled at 4°C for 1 h, infected with NDV (100 PFU), and simultaneously supplemented with 9-butyl-harmol (5 μM) at 4°C for 1 h. Unbound viruses and residual compounds were washed three times with prechilled PBS. (ii) DF-1 cells were incubated with 9-butyl-harmol (5 μM) at 37°C for 1 h, prechilled at 4°C for 1 h prior to infection with NDV (100 PFU) at 4°C for another 1 h. The DF-1 cells were then covered with medium-containing methylcellulose (1%). Plaques were visualized and counted via staining with crystal violet after 72 h.

### Penetration assay.

DF-1 cells were prechilled at 4°C for 1 h and infected with NDV (MOI = 5). Then, the cells were incubated with 9-butyl-harmol (5 μM) or DMSO. At 4 h postincubation, viral proteins were detected via immunofluorescence or Western blot.

### Time-of-addition assay.

A time of drug-addition assay was performed as previously reported to determine the mode of its activity (9). DF-1 cells were infected with NDV (MOI = 0.01). 9-butyl-harmol was added at a concentration of 5 μM at different time points either before infection or postinfection: 24 h before infection, 12 h before infection, or 0, 2, 4, 6, 8, or 12 h postinfection. At 24 h postinfection, the supernatant was collected, and the viral yield was assessed via plaque assay.

### Western blot analysis.

Whole-cell lysates were isolated with RIPA (Solarbio) or M-PER (Thermo Fisher Scientific) lysis buffer supplemented with protease and phosphatase inhibitor cocktail (TargetMol). Cell debris were removed via centrifugation at 12,000 rpm at 4°C for 10 min. The cell samples were then heated along with 5× protein loading buffer at 96°C for 5 min.

The following antibodies were applied. HSP90 polyclonal antibody (Proteintech; catalog number 13171-1-AP) was used at a 1:1,000 dilution. HSP90 monoclonal antibody (Proteintech; catalog number 60318-1-Ig) was used at a 1:1,000 dilution. HSP90AB1 polyclonal antibody (Proteintech; catalog number 11405-1-AP) was used at a 1:1,000 dilution. Beta catenin polyclonal antibody (Proteintech; catalog number 51067-2-AP) was used at a 1:2,000 dilution. NDV NP monoclonal antibody (gifted by Chan Ding) was used at a 1:1,000 dilution. Beta Actin monoclonal antibody (Proteintech. catalog number 66009-1-Ig) was used at a 1:2,000 dilution. GSK-3β (D5C5Z) XP rabbit MAb (Cell signaling technology. catalog number 12456) was used at a 1:1,000 dilution. HA-Tag (C29F4) rabbit MAb (Cell signaling technology. catalog number 3724) was used at a 1:2,000 dilution. DYKDDDDK Tag (9A3) mouse MAb (Cell Signaling Technology. catalog number 8146) was used at a 1:1,500 dilution. MYC-Tag antibody (Proteintech. catalog number 16286-1-AP) was used at a 1:1,000 dilution. Histone-H3 polyclonal antibody (Proteintech. catalog number 17168-1-AP) was used at a 1:2,000 dilution. Anti-rabbit IgG, HRP-linked antibody (Cell signaling technology. catalog number 7074) was used at a 1:2,000 dilution. Anti-mouse IgG, HRP-linked antibody (Cell signaling technology. catalog number 7076) was used at a 1:2,000 dilution.

### Drug affinity responsive target stability (DARTS).

DARTS was performed as previously reported with some modifications (25, 45). Briefly, DF-1 cells were lysed with ice-cold M-PER reagent supplemented with protease inhibitor cocktail and phosphatase inhibitor cocktail on ice for 10 min. The cell lysates were centrifuged for 20 min at 12,000 rpm at 4°C, and the supernatants were mixed with 10× TNC buffer. The mixtures were incubated with DMSO or 9-butyl-harmol (200 μM) at 4°C overnight and added with 0, 50, 100, or 200 ng of pronase (Sigma-Aldrich, USA) at room temperature. At 10 min postincubation, 10× protease inhibitor cocktail was added to stop the digestion. The samples were added with 5× SDS-PAGE loading buffer and heated at 70°C for 10 min. Subsequently, the resulting samples were subjected to a Western blot analysis.

### Cellular thermal shift assay (CETSA).

CETSA was performed as previously reported, with some modifications (46, 47). Briefly, DF-1 cells were lysed with ice-cold M-PER reagent supplemented with protease inhibitor cocktail and phosphatase inhibitor cocktail on ice for 10 min. The supernatants were incubated overnight with DMSO or 9-butyl-harmol (200 μM) at 4°C. After incubation, the samples were divided into 30 μL aliquots and heated for 5 min at the indicated temperature in a PCR machine. This was followed by 10 min of cooling on ice. 5× SDS-PAGE loading buffer was added to the samples and heated at 70°C for 10 min. Subsequently, the resulting samples were subjected to a Western blot analysis.

### Molecular docking.

Molecular docking was conducted using the Autodock Vina and AutodockTools-1.5.6 (48). The crystal structures of GSK-3β (PDB ID: 5KPM) and HSP90β (PDB ID: 5FWK) were obtained from the Protein Data Bank (http://www.pdb.org). The interactions were analyzed using the PyMol and Discovery Studio Visualizer.

### Transcriptome analysis.

DF-1 cells were treated with DMSO or 9-butyl-harmol (10 μM) for 24 h (3 biological replicates in each group). RNAiso Plus (1 mL; TaKaRa) was added to each sample, and the total RNA was extracted, following the manufacturer’s instructions. The quantity and quality of the RNA samples were measured using a NanoPhotometer spectrophotometer (Implen, Germany). The integrity of each RNA sample was assessed with an Agilent 2100 Bioanalyzer (Agilent Technologies, USA). Six RNA samples were used to generate the cDNA libraries. The sequencing of the libraries was performed using an Illumina HiSeq 2500 sequencer (Illumina, USA). All of the clean reads were aligned to the reference genome using Hisat2 v2.0.5. The differential expression genes (DEGs) analysis of two groups was performed using the DESeq2 R package (1.16.1). The data sets for the transcriptome analysis (six samples) were deposited in the Gene Expression Omnibus (GEO) database under accession numbers GSM6805713 to GSM6805718.

### Immunofluorescence microscopy.

The cells were washed three times with PBS and were fixed in 4% paraformaldehyde, permeabilized with 0.5% Triton X-100 for 20 min, blocked in PBST containing 5% BSA for 1 h at 37°C, and then incubated with primary antibody at 37°C for 1 h. Then, the cells were incubated with a second antibody at 37°C for 1 h. Actin filaments were stained with TRITC-phalloidin (2 μg/mL) for 30 min at 37°C. Cell nuclei were stained with DAPI (1 μg/mL) for 10 min. The cells were visualized using a confocal laser scanning microscope (LEICA TCS SP8, Germany).

HA-Tag (C29F4) rabbit MAb (Cell signaling technology. catalog number 3724) was used at a 1:200 dilution. DYKDDDDK Tag (9A3) mouse MAb (Cell Signaling Technology. catalog number 8146) was used at a 1:200 dilution. MYC-Tag antibody (Proteintech. catalog number 16286-1-AP) was used at a 1:200 dilution. NDV NP monoclonal antibody (gifted by Chan Ding) was used at a 1:100 dilution. Beta catenin polyclonal antibody (Proteintech. catalog number 51067-2-AP) was used at a 1:100 dilution. Alexa Fluor 594 AffiniPure Goat Anti-Rabbit IgG (H+L) (Yeasen catalog number 33112ES60) was used at a 1:200 dilution. Alexa Fluor 594 AffiniPure Goat Anti-Mouse IgG (H+L) (Yeasen catalog number 33212ES60) was used at a 1:200 dilution. Alexa Fluor 488 AffiniPure Goat Anti-Mouse IgG (H+L) (Yeasen catalog number 33206ES60) was used at a 1:200 dilution.

### Coimmunoprecipitation.

DF-1 cells were transfected with plasmids of viral proteins (NP, P, or L) together with the plasmids of HSP90 (HSP90AA1 or HSP90AB1) using the Turbofect transfection reagent (Thermo Fisher Scientific). At 48 h posttransfection, the cells were harvested and lysed with lysis buffer containing a protease inhibitor cocktail and a phosphatase inhibitor cocktail. The cell lysate was precleared and incubated with the indicated antibody or an isotype-control with rotation for 4 h. Subsequently, antibody-protein complexes were purified with protein A/G Plus-Agarose (Santa Cruz Biotechnology) overnight at 4°C. The mixture was centrifuged at 3,000 rpm for 3 min at 4°C. The beads were washed three times with lysis buffer and were resuspended in lysis buffer and 5 × SDS-PAGE loading buffer. This was followed by heating at 96°C for 5 min. Finally, the resulting samples were subjected to a Western blot analysis.

### Luciferase reporter assay.

DF-1 cells were transfected with reporter plasmids, such as TOPFlash, FOPFlash, or IFN-β-luc, along with the internal control plasmid pRL-TK using the Turbofect transfection reagent (Thermo Fisher Scientific). At 24 h posttransfection, the cells were treated with compounds or infected with NDV, as indicated, for an additional 24 h. Subsequently, the cells were harvested for the quantification of the luciferase activity with a dual-specific luciferase assay kit (catalog number RG027, Beyotime), according to the manufacturer’s instructions.

### Plasmids and transfection.

pCAGGS-myc-L was obtained from Shanhui Ren (Lanzhou Veterinary Research Institute). pcDNA-HA-NP, pcDNA-HA-P, and pRL-TK were obtained from Wenbin Wang (Shandong Academy of Agricultural Science). M50 Super 8× TOPFlash (catalog number 12456) and M51 Super 8× FOPFlash (catalog number 12457) were purchased from Addgene (USA). The chicken IFN-β-luc was preserved in our laboratory. The β-catenin, HSP90AA1, and HSP90AB1 genes of chicken were amplified and cloned into the pCAGGS plasmid with fused N-terminal tags (β-catenin, HA tag; HSP90AA1/HSP90AB1, Flag tag; HSP90AA1, HA tag). The primers that were used in this study are listed in Table 4.

The transfections were performed according to the protocol of the Turbofect transfection reagent (Thermo Fisher Scientific). For the plasmid transfections, 5 × 10^5^ DF-1 cells were seeded onto 12-well plates and were then transfected with the indicated amounts of plasmids. For siRNA transfection, 5 × 10^5^ DF-1 cells were seeded onto 12-well plates and then transfected with the siRNA at the concentration of 100 nM. The siRNAs for β-catenin, HSP90AA1, and HSP90AB1 were designed and synthesized by RiboBio (China). The siRNAs that were used in this study are listed in Table 5.

**TABLE 5:** The sequences of the siRNAs used in this study

Gene name	Sequence
siNC	UUCUCCGAACGUGUCACGUTT
siHSP90AA1-1	CAGATGACATCACCAATGA
siHSP90AA1-2	ACAAAGCAAGATCCTTAAA
siHSP90AB1-1	CCTGAGTACCTGAACTTCA
siHSP90AB1-2	GCAGAAGAGTATCTACTAT

### Statistical analysis.

The protein bands of the immunoblots were quantified via densitometry using ImageJ. The data are representative of means ± standard deviations (SD). Statistical analysis was done via two-tailed Student’s *t* tests, using data obtained from three independent experiments. Statistical significance: ns, not significant; *, *P < *0.05; **, *P < *0.01; and ***, *P < *0.001.
