# Supplementary material for: 9-Butyl-Harmol Exerts Antiviral Activity against Newcastle Disease Virus through Targeting GSK-3β and HSP90β
Source: J Virol. 2023 Mar 6;97(3):e01984-22. doi: 10.1128/jvi.01984-22 (PMC10062145; doi:10.1128/jvi.01984-22)
Supplement: Supplemental file 1 — Tables S1 and S2 and Fig. S1 to S7. Download jvi.01984-22-s0001.docx, DOCX file, 3.4 MB [file jvi.01984-22-s0001.docx]

Supplementary Material

**9-Butyl-Harmol Exerts Antiviral Activity against Newcastle Disease Virus through Targeting GSK-3β and HSP90β**

Chongyang Wang,^a,b^ Ting Wang,^a^ Ruochen Hu,^a^ Liuyuan Duan,^a^ Qili Hou,^a^ Yu Han,^a^ Jiangkun Dai,^b^ Wenbin Wang,^c^ Shanhui Ren,^d^ Haijin Liu,^a^ Xinglong Wang,^a^ Sa Xiao,^a^ Na Li,^e^ Junru Wang,^b^ Zengqi Yang^a^

^a^ College of Veterinary Medicine, Northwest A&F University, Yangling, China

^b^ College of Chemistry and Pharmacy, Northwest A&F University, Yangling, China

^c^ Poultry Institute, Shandong Academy of Agricultural Science, Jinan, China

^d^ State Key Laboratory of Veterinary Etiological Biology, Lanzhou Veterinary Research Institute, Chinese Academy of Agricultural Sciences, Lanzhou, China

^e^ Instrumental Analysis Center, Xi'an Jiaotong University, Xi’an, China

Address correspondence to Zengqi Yang, yzq8162@126.com (biology), or Junru Wang, wangjunru@nwsuaf.edu.cn (chemistry).

**Table S1** and **S2** provide the structures of *β*-carboline derivatives used in this study.

**Figure S1** and **S2** provide the results of primary screening of *β*-carboline monomers and dimers.

**Figure S3** provides the antiviral activity of *β*-carboline derivatives against paramyxoviruses including PPRV and CDV.

**Figure S4** provides the antiviral effect of 9-butyl-harmol against NDV.

**Figure S5** provides the effect of 9-butyl-harmol on the expression of ISGs during NDV infection.

**Figure S6** displays the effect of NDV infection on the expression level of HSP90.

**Figure S7** displays the interference efficiency of siRNAs targeting HSP90.

**Table S1**

The numbers and structures of *β*-carboline monomers

| **No.** | Structure | No. | Structure |
| --- | --- | --- | --- |
| **1** | **** | **2** | **** |
| **3** | **** | **4** | **** |
| **5** | **** | **6** | **** |
| **7** | **** | **8** | **** |
| **9** | **** | **10** | **** |
| **11** | **** | **12** | **** |
| **13** | **** | **14** | **** |

**Table S2**

The numbers and structures of *β*-carboline dimers

| **No**. : R | Structure |
| --- | --- |
| **15**：Butane-1,4-diyl  **16**：Pentane-1,5-diyl  **17**：Hexan-1,6-diyl  **18**：Octan-1,8-diyl  **19**：*p*-Xylene-α,α’-diyl |  |
| **20**：Butane-1,4-diyl  **21**：Pentane-1,5-diyl  **22**：Hexan-1,6-diyl  **23**：Octan-1,8-diyl  **24**：*p*-Xylene-α,α’-diyl |  |
| **25**：Butane-1,4-diyl  **26**：Pentane-1,5-diyl  **27**：Hexan-1,6-diyl  **28**：Octan-1,8-diyl  **29**：*p*-Xylene-α,α’-diyl |  |
| **30**：Butane-1,4-diyl  **31**：Pentane-1,5-diyl  **32**：Hexan-1,6-diyl  **33**：Octan-1,8-diyl |  |
| **34**：Butane-1,4-diyl  **35**：Pentane-1,5-diyl  **36**：Hexan-1,6-diyl  **37**：Octan-1,8-diyl |  |
| **38**：Butane-1,4-diyl  **39**：Pentane-1,5-diyl  **40**：Hexan-1,6-diyl  **41**：Octan-1,8-diyl |  |
| **42**：Butane-1,4-diyl  **43**：Pentane-1,5-diyl  **44**：Hexan-1,6-diyl  **45**：Octan-1,8-diyl |  |
| **46**：Butane-1,4-diyl  **47**：Pentane-1,5-diyl  **48**：Hexan-1,6-diyl  **49**：Octan-1,8-diyl |  |
| **50**：Butane-1,4-diyl  **51**：Pentane-1,5-diyl  **52**：Hexan-1,6-diyl  **53**：Octan-1,8-diyl |  |


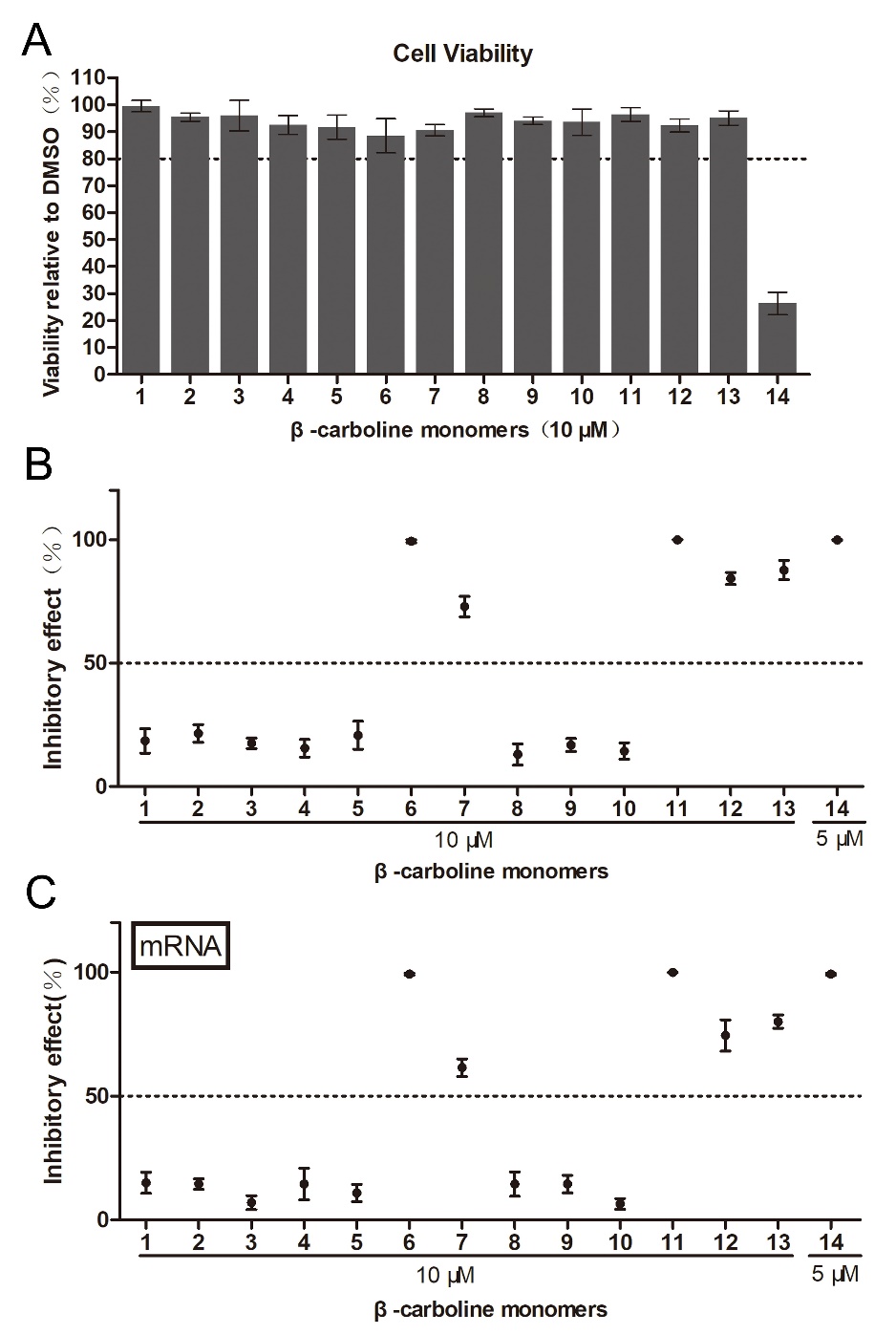


**Figure S1** **Primary screening of *β*-carboline monomers with antiviral activities.**

(A) Cell viability was assessed by CCK-8 in DF-1 cells treated with *β*-carboline monomers at 48 h post-incubation.

(B, C) DF-1 cells were infected with F48E9 (0.01 MOI). After adsorption (1 h), cells were covered with DMEM containing *β*-carboline monomers at indicated concentrations. At 24 h post-infection, the virus yield in the supernatant was measured by plaque assay (B). The relative mRNA expression was measured by qRT-PCR (C).


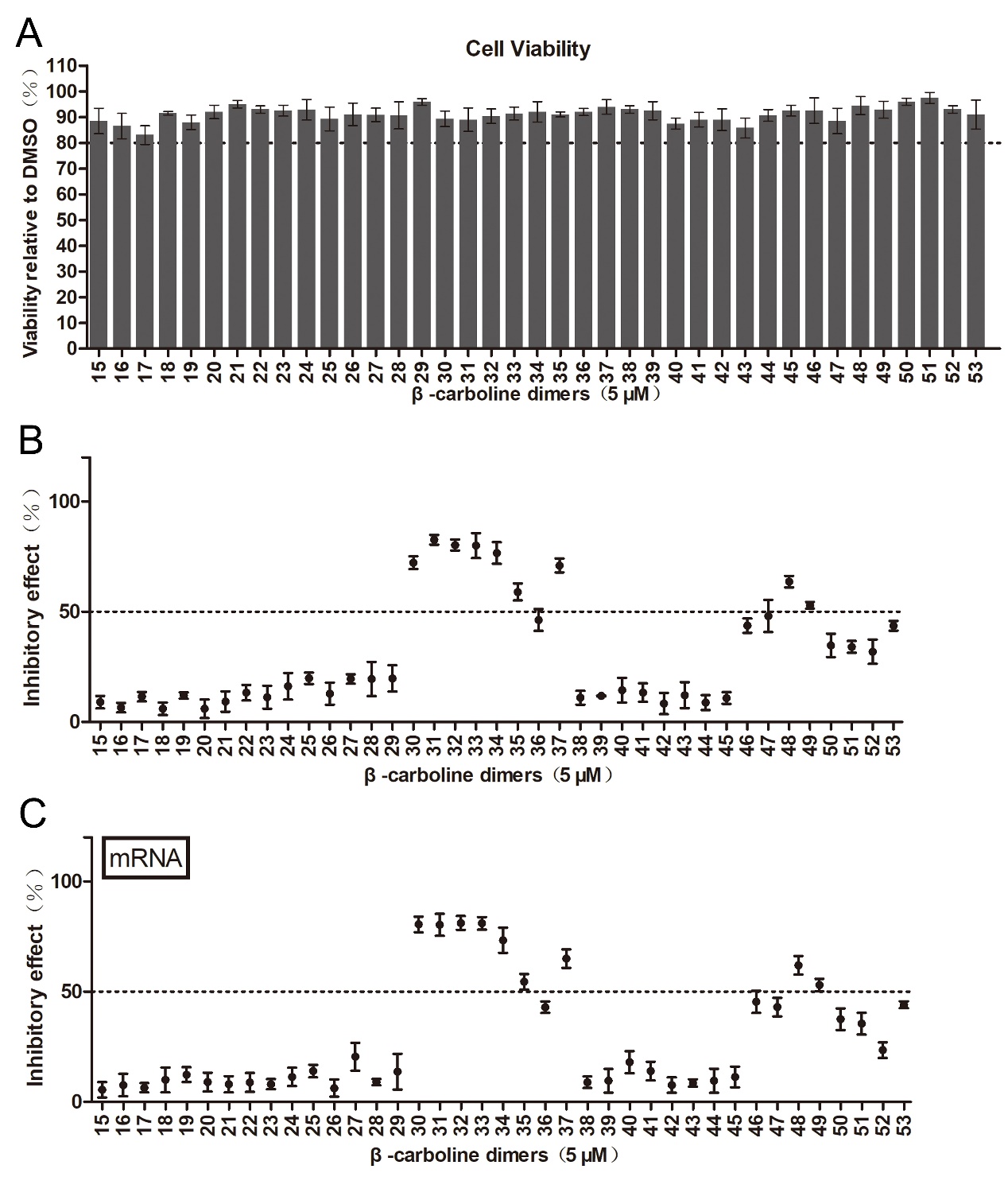


**Figure S2 Primary screening of *β*-carboline dimers with antiviral activities.**

(A) Cell viability was assessed by CCK-8 in DF-1 cells treated with *β*-carboline dimers at 48 h post-incubation.

(B, C) DF-1 cells were infected with F48E9 (0.01 MOI). After adsorption (1 h), cells were covered with DMEM containing *β*-carboline dimers (5 μM). At 24 h post-infection, the virus yield in the supernatant was measured by plaque assay (B). The relative mRNA expression was measured by qRT-PCR (C).


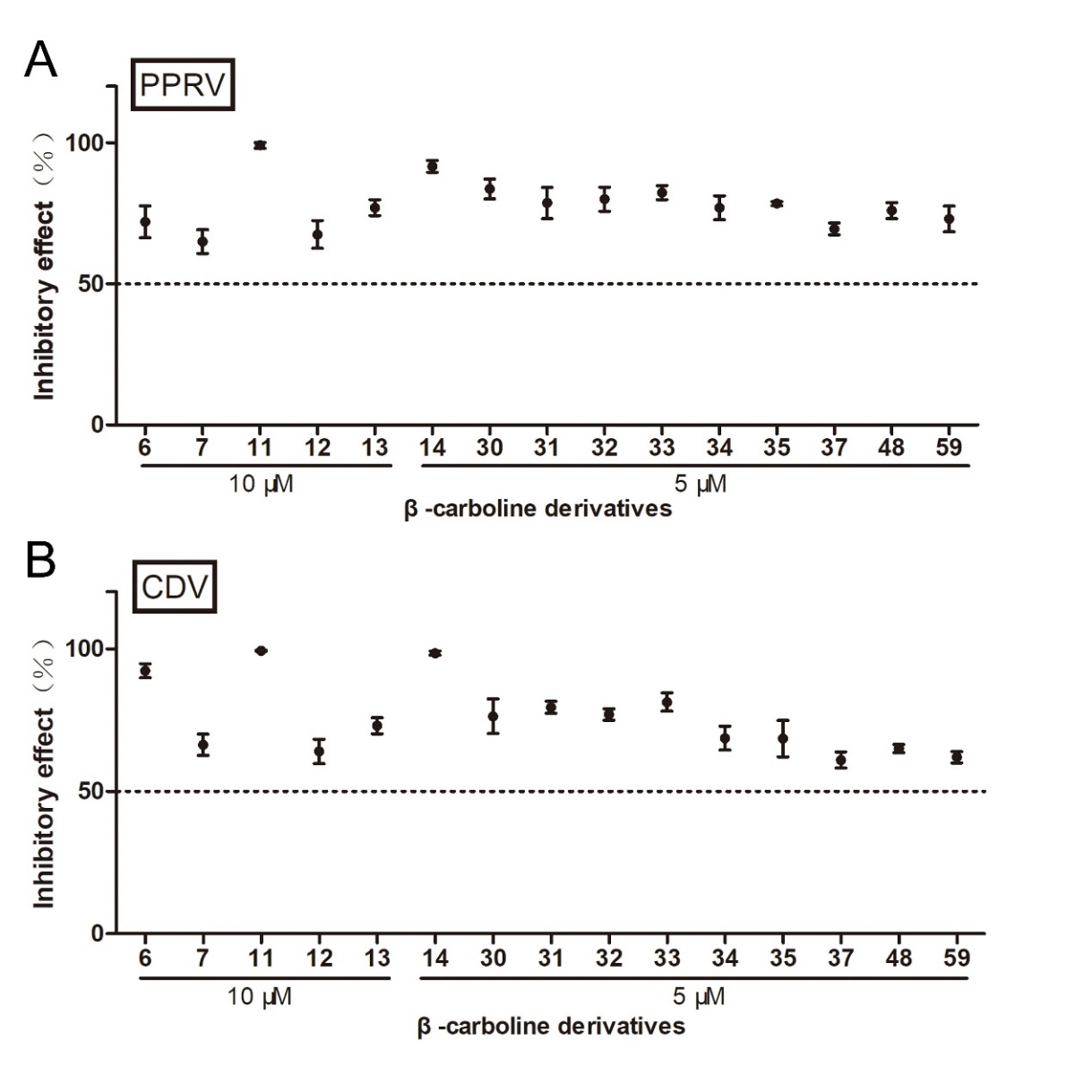


**Figure S3** **The antiviral activity of *β*-carboline derivatives against PPRV and CDV.**

(A, B) Vero cells were infected with PPRV (A) or CDV (B) and treated with DMSO or *β*-carboline derivatives. At 24 h post-infection, viral mRNA was assessed by qRT-PCR.


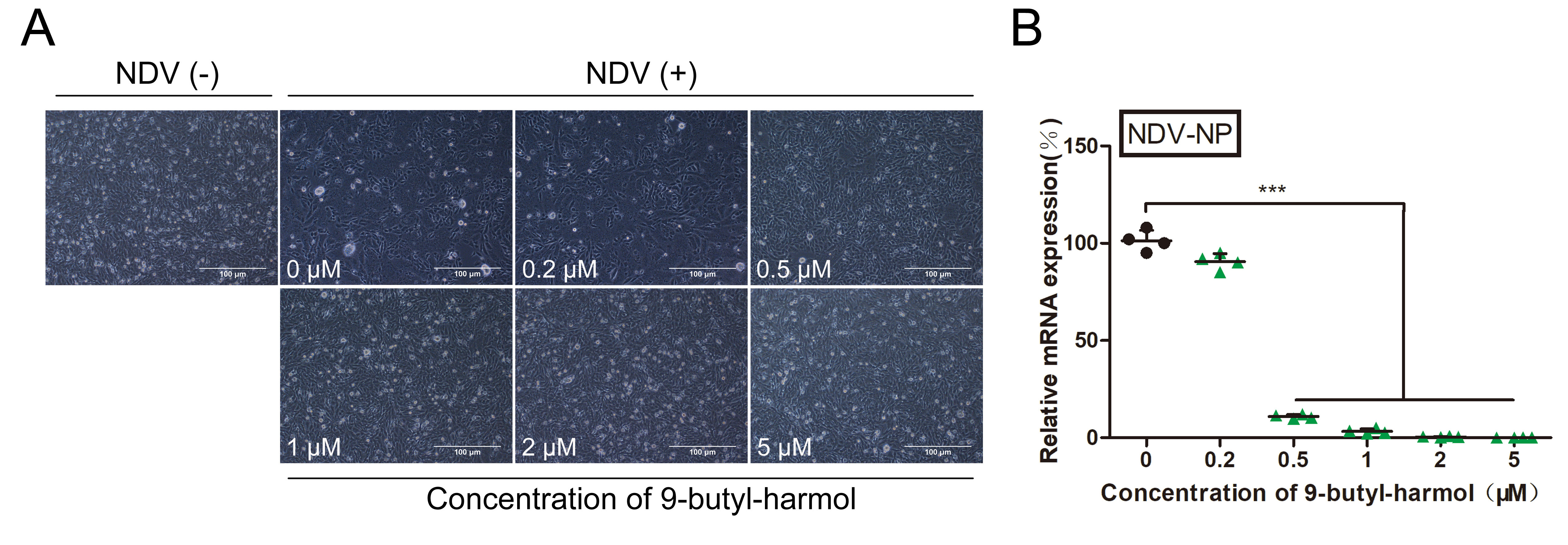


**Figure S4** **The antiviral effect of 9-butyl-harmol against NDV.**

(A, B) DF-1 cells were infected with F48E9 (0.01 MOI), then the medium was changed for fresh medium containing 9-butyl-harmol, followed by a 24 h incubation. The cytopathic effect was photographed (A). The viral mRNA was assessed by qRT-PCR (B). Mean values ± SDs are shown (n = 4). Significance assessed with two-tailed, unpaired Student’s *t*-test, ****P* < 0.001.


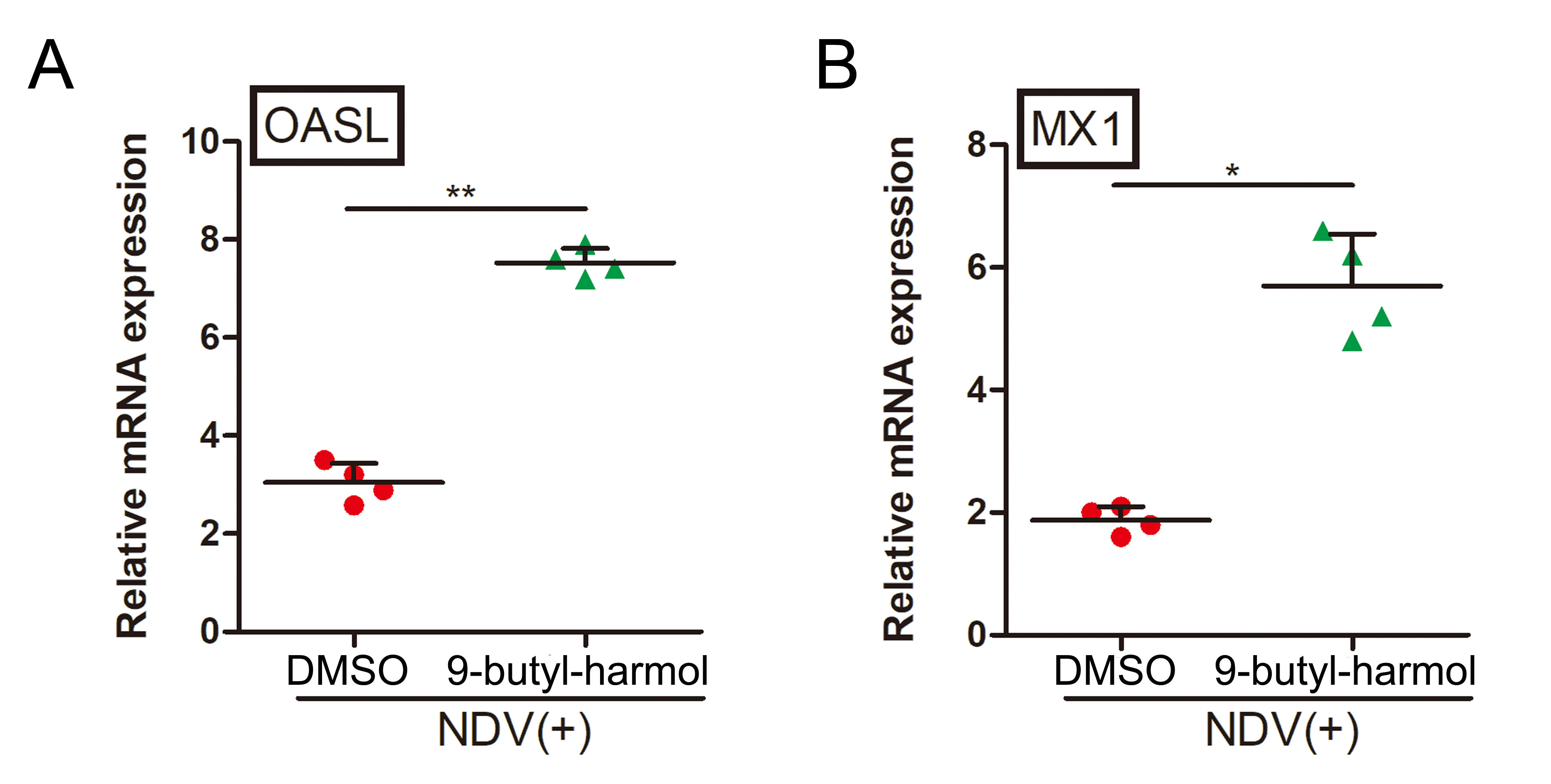


**Figure S5 9-butyl-harmol promotes the expression of ISGs during NDV infection.**

(A, B) 9-butyl-harmol promotes the expression of IFN-β during NDV infection. DF-1 cells were infected with NDV (0.01 MOI) and then incubated with DMSO or 9-butyl-harmol (5 μM). At 24 h post-incubation, cells were harvested to quantify the expression of OASL (left) and MX1 (right) by qRT-PCR. Mean values ± SDs are shown (n = 4). Significance assessed with two-tailed, unpaired Student’s *t*-test, **P* < 0.05, ***P* < 0.01.


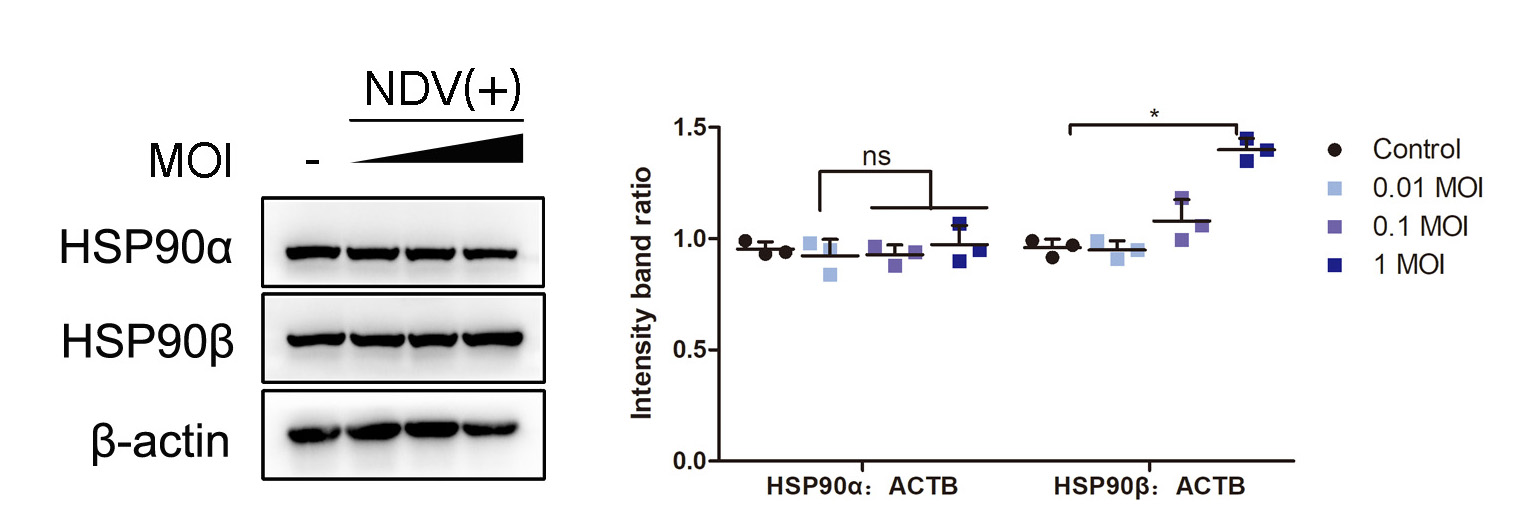


**Figure S6 The effect of NDV infection on the expression level of HSP90.**

DF-1 cells were infected with NDV (0.01, 0.1 or 1 MOI). At 12 h post-infection, cells were harvested to assess the expression of protein by Western blot (left). The relative quantification of the target protein level was analyzed by ImageJ (right). Mean values ± SDs are shown (n = 3). Significance assessed with two-tailed, unpaired Student’s *t*-test, ns, not significant, **P* <0.05.


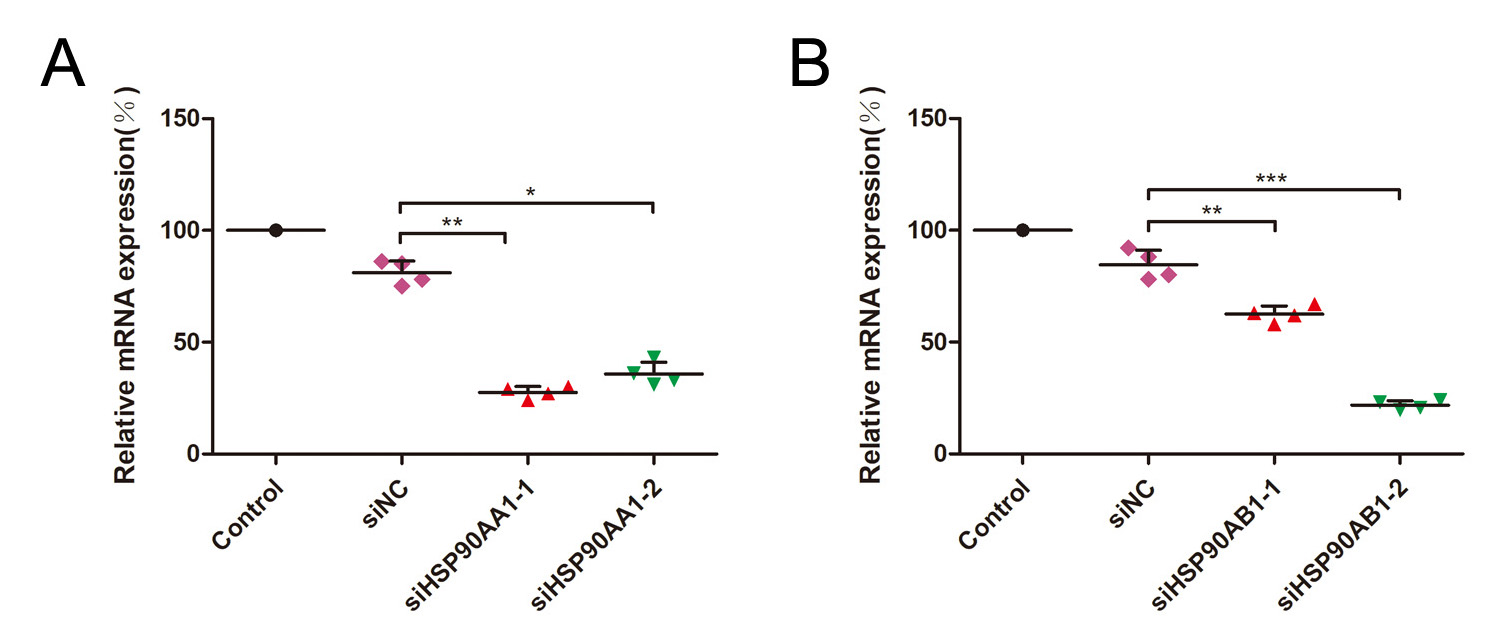


**Figure S7 Interference efficiency of siRNAs targeting HSP90.**

(A, B) DF-1 cells were transfected with siRNAs targeting HSP90AA1 (A) or HSP90AB1 (B). At 48 h post-transfection, cells were harvested to assess the interference efficiency by qRT-PCR. Mean values ± SDs are shown (n = 4). Significance assessed with two-tailed, unpaired Student’s *t*-test, **P* <0.05, ***P* < 0.01, ****P* < 0.001.
